# Supplementary material for: Programme and policy perspectives towards a tobacco-free generation in India: findings from a qualitative study
Source: BMJ Open. 2023 Jul 7;13(7):e067779. doi: 10.1136/bmjopen-2022-067779 (PMC10335429; doi:10.1136/bmjopen-2022-067779)
Supplement: Supplementary data [file bmjopen-2022-067779supp001.pdf]

Supplementary Table: An overview of qualitative data collection method, participants and themes

**Themes**	**Stakeholders at National level**	**Stakeholders at state level**	**Stakeholders at district level**	**Stakeholders at village level**
Existing adolescent specific tobacco control programmes to prevent tobacco uptake among adolescents	✓	✓	✓	
Existing adolescent specific tobacco control policies to prevent tobacco uptake among adolescents	✓	✓	✓	
Perception on extent of program and policy implementation	✓	✓	✓	✓
Perception on designing and implementing adolescent specific tobacco control policies	✓	✓	✓	✓
Barriers/gaps for successful implementation of adolescent specific tobacco control policies and programmes	✓	✓	✓	✓
Facilitators for successful implementation of adolescent specific tobacco control policies and programmes	✓	✓	✓	✓
Recommended strategies to prevent and reduce the use of tobacco among adolescents	✓	✓	✓	✓
Need for programme evaluation and research priorities	✓	✓	✓	

Bassi S, *et al. BMJ Open* 2023; 13:e067779. doi: 10.1136/bmjopen-2022-067779
