# Supplementary material for: Programme and policy perspectives towards a tobacco-free generation in India: findings from a qualitative study
Source: BMJ Open. 2023 Jul 7;13(7):e067779. doi: 10.1136/bmjopen-2022-067779 (PMC10335429; doi:10.1136/bmjopen-2022-067779)
Supplement: Author's manuscript [file bmjopen-2022-067779.draft_revisions.pdf]

BMJ Open

BMJ Open is committed to open peer review. As part of this commitment we make the peer review history of every article we publish publicly available.

When an article is published we post the peer reviewers' comments and the authors' responses online. We also post the versions of the paper that were used during peer review. These are the versions that the peer review comments apply to.

The versions of the paper that follow are the versions that were submitted during the peer review process. They are not the versions of record or the final published versions. They should not be cited or distributed as the published version of this manuscript.

BMJ Open is an open access journal and the full, final, typeset and author-corrected version of record of the manuscript is available on our site with no access controls, subscription charges or pay-per-view fees (http://bmjopen.bmj.com).

If you have any questions on BMJ Open's open peer review process please email info.bmjopen@bmj.com

Program and Policy Perspective Towards a Tobacco-Free Generation: A Qualitative Study

Journal:	*BMJ Open*
Manuscript ID	bmjopen-2022-067779
Article Type:	Original research
Date Submitted by the Author:	29-Aug-2022

Complete List of Authors:	Bassi, Shalini; Public Health Foundation of India, Health Promotion Division; HRIDAY,<br>Arora, Monika; HRIDAY; Public Health Foundation of India, Health Promotion Division<br>Thapliyal, Nishibha; Public Health Foundation of India, Health Promotion Division<br>Kulkarni, Muralidhar; Kasturba Medical College Manipal, Community Medicine<br>Bhagawath, Rohith; Kasturba Medical College Manipal, Community Medicine<br>Bogdanovica, Ilze; University of Nottingham, School of Medicine<br>Kamath, Veena; Kasturba Medical College Manipal, Community Medicine<br>Britton, John; University of Nottingham, Epidemiology and Public Health<br>Bains, Manpreet; University of Nottingham, School of Medicine

Keywords:	QUALITATIVE RESEARCH, PUBLIC HEALTH, Health policy < HEALTH SERVICES ADMINISTRATION & MANAGEMENT

SCHOLARONE™
Manuscripts

I, the Submitting Author has the right to grant and does grant on behalf of all authors of the Work (as defined in the below author licence), an exclusive licence and/or a non-exclusive licence for contributions from authors who are: i) UK Crown employees; ii) where BMJ has agreed a CC-BY licence shall apply, and/or iii) in accordance with the terms applicable for US Federal Government officers or employees acting as part of their official duties; on a worldwide, perpetual, irrevocable, royalty-free basis to BMJ Publishing Group Ltd ("BMJ") its licensees and where the relevant Journal is co-owned by BMJ to the co-owners of the Journal, to publish the Work in this journal and any other BMJ products and to exploit all rights, as set out in our licence.

The Submitting Author accepts and understands that any supply made under these terms is made by BMJ to the Submitting Author unless you are acting as an employee on behalf of your employer or a postgraduate student of an affiliated institution which is paying any applicable article publishing charge ("APC") for Open Access articles. Where the Submitting Author wishes to make the Work available on an Open Access basis (and intends to pay the relevant APC), the terms of reuse of such Open Access shall be governed by a Creative Commons licence – details of these licences and which Creative Commons licence will apply to this Work are set out in our licence referred to above.

Other than as permitted in any relevant BMJ Author's Self Archiving Policies, I confirm this Work has not been accepted for publication elsewhere, is not being considered for publication elsewhere and does not duplicate material already published. I confirm all authors consent to publication of this Work and authorise the granting of this licence.

For peer review only

Title: Program and Policy Perspective Towards a Tobacco-Free Generation: A Qualitative Study

Authors: Shalini Bassi[1,2]*, Monika Arora[1,2], Nishibha Thapliyal[2], Muralidhar M Kulkarni[3], Rohith Bhagawath[3], Ilze Bogdanovica[4], Veena G Kamath[3], John Britton[4], Manpreet Bains[4]

[1] Health Related Information Dissemination Amongst Youth (HRIDAY)

[2] Public Health Foundation of India (PHFI)

[3] Manipal Academy of Higher Education

[4] University of Nottingham

Corresponding Author*

Ms. Shalini Bassi,

Senior Research Scientist, Health Promotion Division, Public Health Foundation of India (PHFI), Gurgaon, Haryana, 122002, India. shalini.bassi@phfi.org

&

Consultant-HRIDAY

N-25, Second Floor, Green Park Extension, New Delhi – 110016, India. shalini@hriday-shan.org

Author Information

Ms. Shalini Bassi, Health Promotion Division, Public Health Foundation of India (PHFI), Gurugram, India. & HRIDAY, New Delhi, India. shalini.bassi@phfi.org

Dr. Monika Arora, HRIDAY, New Delhi, India & Health Promotion Division, Public Health Foundation of India (PHFI), Gurgramn, Haryana, India. monika@hriday-shan.org

Dr. Nishibha Thapliyal, Health Promotion Division, Public Health Foundation of India (PHFI), Gurugram, India, nishibhathapliyal@gmail.com

For peer review only - http://bmjopen.bmj.com/site/about/guidelines.xhtml

Dr. Muralidhar M Kulkarni, Community Medicine, Kasturba Medical College Manipal, Udupi, Karnataka, India. murali.kulkarni@manipal.edu

Dr. Veena Ganesh Kamath, Community Medicine, Kasturba Medical College Manipal, Udupi, Karnataka, India. veenak@manipal.edu

Dr. Rohith Bhagawath, Community Medicine, Kasturba Medical College Manipal, Manipal Academy of Higher Education, Udupi, Karnataka, India. rohit.bhagawath@manipal.edu

Dr. Ilze Bogdanovica, School of Medicine, University of Nottingham, Nottingham, United Kingdom. Ilze.Bogdanovica@nottingham.ac.uk

Prof. John Britton, Emeritus Professor; Director, UK Centre for Tobacco & Alcohol Studies, University of Nottingham, Division of Epidemiology and Public Health, Nottingham, United Kingdom. John.Britton1@nottingham.ac.uk; j.britton@outlook.com

Dr. Manpreet Bains, School of Medicine, University of Nottingham, Nottingham, United Kingdom. manpreet.bains@nottingham.ac.uk

Word Count – Abstract- 261 words; Main document- 4244

For peer review only - http://bmjopen.bmj.com/site/about/guidelines.xhtml

ABSTRACT

Objective: This study explored multi-stakeholder perspectives on existing adolescent specific tobacco control policies and programmes, barriers to and facilitators, lessons and recommendations for their successful implementation, as well as their strengthening to advance India's transition towards a tobacco-free generation.

Design: Qualitative research

Setting: Semi-structured interviews were conducted with key officials at the national (India), state (Karnataka), district (Udupi) and village level. Interviews were audio-recorded, transcribed verbatim and analysed thematically.

Participants: We conducted 38 semi-structured interviews across national (n=9), state (n=9), district (n=14) and village (n=6) levels.

Results: Our findings emphasized the need to amend and strengthen, existing provisions of Tobacco Control Law (2003) especially in and around schools (section 6a & 6b) and the development of a tobacco monitoring and surveillance application for educational institutes. Findings also suggested increasing the legal age of access to tobacco from 18 to 21 years. Considering the magnitude of smokeless tobacco use in India, policies to address this problem were considered a priority. The need of strict monitoring of E-cigarette ban on OTT platforms has been stressed. Engaging adolescents meaningfully and co-creation of interventions was also emphasized. Combining both an inter-sectoral and whole-societal approach to prevent tobacco use among adolescents was also advocated. While drafting and implementing a comprehensive national tobacco control policy the findings revealed the need to adopt a vision towards a tobacco-free generation.

For peer review only - http://bmjopen.bmj.com/site/about/guidelines.xhtml

Conclusion: Our findings highlight a need to strengthen the existing and developing new tobacco control programmes and policies focusing adolescents. The findings revealed the need to adopt a vision towards a tobacco-free future generation and replicate such models globally.

Strengths and limitations of this study

- This study captures a wide range of perspective from representatives from different levels of governance (national/state/district/village).
- The study findings provide recommendations for strengthening the enforcement of existing and development of new policies towards the ultimate goal of reaching a tobacco-free generation or tobacco endgame for India as well as replicate such models globally.
- We have captured the perspective of various stakeholders at state, national, district and village level other than adolescents, thus the beneficiary perspective (adolescents) on tobacco control policies is missing. However, their perspective will be presented through a separate research paper carried out to understand the perception towards the current policies on tobacco control among adolescents and recommendations to address the tobacco use behaviour among them.
- The sampling was one of the limitations of our study as the participants for the in-depth interviews were not randomly selected, hence limiting the generalizability of the findings.

For peer review only

BACKGROUND

Tobacco use is a leading risk factor associated with several diseases, premature death and disability. It is a major threat to developing and low/middle income countries such as India, where tobacco use has reportedly led to 1.35 million annual deaths.[1,2] Furthermore, of all deaths related to non-communicable diseases in India in 2019, approximately 1.08 million deaths (17.7%) were due to tobacco use.[3] The prevalence of tobacco use among adults in India has declined from 34% to 28% between 2010 (GATS-1)[4] and 2017 (GATS-2)[5] and among school going students aged 13-15 years from 16.9% (GYTS-1, 2003)[6] to 8.5% (GYTS-4, 2019).[7] The majority of adult tobacco users initiate use during adolescence[8] often leading to lifelong use, with low quit rates.[9] Hence, prevention of tobacco uptake among children and adolescents is of paramount importance for reducing harms caused by tobacco use.

The Government of India (GoI) enforced the Cigarettes and Other Tobacco Products (Prohibition of Advertisement and Regulation of Trade and Commerce, Production, Supply and Distribution) Act (COTPA) in 2003, to prevent uptake of tobacco use by children and adolescents as one of its objectives.[10] Section 6a of COTPA stipulates prohibition of the sale of tobacco products to and by minors; and Section 6b mandates, prohibition on the sale of tobacco products around 100 yards of educational institutions.[10] India also launched the School Programme as part of the 'National Tobacco Control Programme'(NTCP) across public and private schools, with an aim to help young people to acquire the knowledge, attitudes and skills to make informed decisions related to tobacco consumption and understand the consequences of tobacco use.[11] To provide momentum to implementation of these provisions, the Ministry of Health and Family Welfare (MoHFW)-Government of India also introduced 'Tobacco-Free Educational Institution' (ToFEI) guidelines[12], for schools, colleges and universities, in both public and private sector, across India.

For peer review only - http://bmjopen.bmj.com/site/about/guidelines.xhtml

These guidelines aim to generate awareness about harmful and long-term effects of tobacco use and create healthy and tobacco-free environment in educational institutes. This was a critical milestone in advancing tobacco control interventions in and around educational institutions. Despite several of these efforts, implementation of tobacco control policies continues to be a challenge.[13] There is a scientific debate on whether investing in youth smoking control and prevention efforts among adolescents is effective, with mixed outcomes.[14] The aim of this paper was to explore the views of multiple stakeholders (including policy-makers, policy-implementers, representatives from civil society, implementation agencies, and other stakeholders) on India's existing tobacco control policies and programmes focusing on adolescents, barriers to and facilitators for their successful implementation, and to provide recommendations for their successful implementation and strengthening.

METHODS

Design

Qualitative, one-to-one, semi-structured interviews were conducted with key officials at the national (India), state (Karnataka), district (Udupi) and village level.

Patient and Public Involvement

Patients or the public were not involved in the design, or conduct, or reporting, or dissemination plans of our research.

Recruitment and sampling

For peer review only - http://bmjopen.bmj.com/site/about/guidelines.xhtml

A purposive sampling strategy was used[15,16] where a list of 51 eligible individuals was drafted by members of the research team (MA, SB, MK), based on professional networks and level of engagement with tobacco control programs at different levels of governance. The interviewees varied in terms of their roles, responsibilities and seniority, to ensure that a wide range of perspectives were captured. Potential participants were approached by SB and RB via e-mail and telephone. A study information sheet and consent form was sent prior, and an interview was arranged at a time convenient for an interviewee.

Data collection and procedure

A semi-structured interview guide was developed to explore participants' views on: adolescent specific tobacco control existing policies and programmes in India, barriers to and facilitators for their successful implementation, as well as recommendations for their successful implementation and strengthening to advance India's transition towards a tobacco-free generation (Supplementary file here with full list of topics). The interview guide was developed by (SB, MB, with inputs from MA, MK, VK) based on the School Programme component delivered as part of NTCP[11], ToFEI guidelines by MoHFW[12], provisions of Indian Tobacco Control Act (2003)[10] specifically addressing prevention of tobacco uptake and use by children and adolescents.

Interviews were conducted between July 2019 and September 2021, in English by trained qualitative researchers. National and state level stakeholder interviews were moderated by SB, while district and village level by MK and RB. Due to the COVID-19 mitigation measures in India (e.g. travel restrictions), national and state level interviews were conducted virtually using Zoom platform (during December 2020-September 2021), while district and village level 'in-

For peer review only - http://bmjopen.bmj.com/site/about/guidelines.xhtml

person' (during July 2019 -March 2020). The participants were asked to review and sign a consent form prior to interview (verbal consent was sought for virtual interviews). The consent process covered permission for data collection, audio-recording of interviews, data to be used in research publications and dissemination whilst maintaining interviewee confidentiality and anonymity.

Data analysis

Interviews were transcribed verbatim by members of the research team. Identifiable data were anonymized and double checked for accuracy by senior members of the research team. Transcripts were analyzed by SB, NT and MB using thematic analysis[17] where both inductive and deductive approaches were adopted. To ensure validity of interpretations[18] each individual familiarized themselves with the data separately which led to the identification of initial codes. Further readings resulted in the development of more substantive themes and sub-themes. Following this, series of virtual calls were organized between SB, NT and MB to discuss preliminary themes which led to a more definitive thematic framework, which was agreed upon and then applied to the remaining data (by NT) using NVivo 12.0.

RESULTS

In total, 38 interviews were conducted, including nine with national level stakeholders, nine with state level stakeholders (Bengaluru, Karnataka), 14 with district (Udupi) level stakeholders and six village level stakeholders. Interviewees included officials from the NTCP, members of tobacco control committee (national, state and district level), representatives from civil society organizations working in tobacco control, health, education, agriculture, food and civil supplies, police, parliamentary affairs, district administration, rural development and panchayati raj (a

For peer review only

system of local self-government of villages in rural India). Interviews ranged from 20 minutes to 111 minutes, with an average of 44 minutes.

Thematic analysis

The major themes generated were: Existing tobacco control policies and programs focusing on adolescents in India; barriers to and facilitators for their successful implementation and recommendations for successful implementation and strengthening adolescent specific tobacco control policies and programmes to advance India's transition towards a tobacco-free generation

Existing tobacco control policy and programme actions focusing on adolescents in India

When referencing to existing efforts, participants commented on barriers/facilitators, extent of program/policy implementation, challenges and future recommendations for effective adolescent tobacco control.

Our findings reported many tobacco control programmes and policies to prevent tobacco uptake among adolescents. The Government of India enacted COTPA to prevent the uptake of tobacco by children and adolescents as one of its objectives. The government has implemented several programmes as part of numerous initiatives, including school and community-based ones, to prevent adolescents from indulging in tobacco use. The interviewees believed school-based programmes or interventions to be an important way to prevent adolescents using tobacco. Most of the interviewees spoke about the 'School Health Program', which forms part of NTCP and a few also mentioned smaller regional or local initiatives such as, 'Yellow line campaign' (wherein, two yellow lines are painted 100 yards away from the boundary wall of an education institute demarking a 'No Tobacco Zone'); the 'Rose Campaign' (where a 'rose' flower was given to petty shop owners selling tobacco and were made aware of its consequences and asked to display boards with tobacco warnings); the 'National Population Education Project' (creating awareness or helping adolescent understand the impact of using tobacco through number of activities, like

For peer review only

poster competitions, memes, folk dance, skits, role play, mono acting etc) and also other community-based activities with involvement of local leaders and formation of 'Taluka level coordination committee' (a group of several villages counted together for administrative purposes) for controlling tobacco in the community.

As part of the NTCP, the participants mentioned the ongoing sensitization activities (sessions/talks/audio-visual clips) for school children, formation of anti-tobacco school committees, making an anti-tobacco pledge in school assembly, organization of anti-tobacco theme-based competitions like drawing, posters, painting, quiz competitions and relay-races. Some even spoke about anti-tobacco message competitions and role play activities, along with annual cultural-day programs in schools, in order to generate awareness on tobacco, among both students and parents. State representative from the Education department mentioned that many school teachers are also invited to be a part of sensitization training organized by the Government which creates awareness about the ill effects of using tobacco in school children, where manuals were reportedly provided to school authorities to aid the process.

> *"We have received a manual, wherein information about cigarette content, activities to be taken by the students for not falling into the trap, is provided. There are many pictures in that, wherein it is displayed about the effects of usage of tobacco, and many more things are there in the manual. There are number of activities and content which creates awareness to students and we have already passed this to school authorities"*

Another component of NTCP spoken about by many interviewees was the ToFEI guidelines on the implementation of tobacco control initiatives among adolescents and young adults, by the

For peer review only - http://bmjopen.bmj.com/site/about/guidelines.xhtml

Ministry of Health and Family Welfare. A national level NGO representative highlighted that the most important indicator in ToFEI, deals with the implementation of section 6b of COTPA around the sale of tobacco near educational institutes:

> *"One of the great outcomes that have come out of this ToFEI program is the section 6b of COTPA which prohibits any shop selling tobacco within 100 yards of any educational institute, right! And the industry makes sure that the shops are there. One of the components in these guidelines makes us report the number of tobacco shops which are around these schools."*

For peer review only

Most interviewees reported poor monitoring and weak enforcement of NTCP and ToFEI guidelines as the primary barrier to its successful implementation. A national level civil society representative stated that, "*there is no mechanism to monitor how many schools are tobacco free in the state"* and another interviewee mentioned limited outreach of the guidelines "*The guidelines are not reaching upto where it has to, like if we talk about educational institutions. The Principals, the administrative authorities of these educational institutions seem to be unaware and they are unaware because nobody has reached out to them"*.

Recommendations for successful implementation and strengthening adolescent specific tobacco control policies and programmes to advance India's transition towards a tobacco-free generation

While talking about the lessons and recommendations for strengthening tobacco control adolescent specific efforts, the majority of the interviewees underlined the continuous need to educate adolescents about the consequences of using tobacco and how the tobacco industry uses

For peer review only - http://bmjopen.bmj.com/site/about/guidelines.xhtml

deceptive tactics to entice the younger generation to take up their products. Using explicit examples, techniques on how the tobacco industry targets young people were highlighted. More use of IEC (Information, Education, Communication) material, videos, short films, awareness campaigns, were suggested, along with the use of newer social media platforms (*Facebook, Twitter, WhatsApp),* due to its popularity among the younger generation.

Many even stated lack of awareness about the ongoing program among members of enforcement agencies (organizations that are responsible for program implementation- education, police department etc.). Hence, stressed the need for targeted sensitization programs for enforcement agencies on the importance of enforcing tobacco control laws.

Interviewees believed school-based interventions to be an important way to prevent adolescents from using tobacco. Suggesting the need for tobacco education in schools, a state representative said to "*Take up important aspects of tobacco education into the curriculum of students wherein they are taught actively that, these are habits that are detrimentally harmful; and the industry is going to try and fool them and to look for industry interference*". Some interviewees also suggested co-creating adolescents driven projects or interventions engaging them in creating awareness among their peers, where an example from a school-based intervention in Delhi schools was referred to:

> *"The MYTRI trial, "if you see this element of peer activism in terms of interventions. So, if you do school-based interventions which are actively involving youth, as a change maker or as advocates, those interventions are more effective".*

A state civil society representative suggested the development of a monitoring tool by the education department, to ensure compliance with ToFEI guidelines.

For peer review only - http://bmjopen.bmj.com/site/about/guidelines.xhtml

"The educational department has to come up with the monitoring tool to assess the level of compliance with ToFEI*. Practically someone from the educational department have to physically verify the compliance. The school may upload the photographs but the physical verification by the concerned officers of educational department and sharing the monthly progress report with the key stakeholders is more helpful to understand the level of compliance."*

A national level interviewee pointed out that the ToFEI guidelines need to be implemented in a proper way, especially in village schools, where teachers must first be oriented, and better collaboration between ministries needs to be addressed. In rural areas, integrating ToFEI guidelines in 'Aadarsh gaon Yojana'(Model village, Rural Development Model)[19] which focuses on village development and includes social, cultural development and social mobilization of the village community; or Clean India Mission (Swaach Bharat Abhiyan)[20] a countrywide campaign initiated by the Government of India to eliminate open defecation and improve solid waste management, was suggested.

To reduce and prevent tobacco use amongst youth, majority of the interviewee emphasized strict enforcement of the COTPA Act in the vicinity of educational institutions. Many even stated that specific amendments to COTPA-2003, especially surrounding educational institutes concerning increasing penalties/fines were needed. The need for strengthening implementation of 6a and 6b of COTPA-2003 was highlighted by the state level Civil Society representative, "*So we have COTPA section 6a, and 6b provision. It says tobacco cannot be sold within 100 yards of any educational institution, so if you implement this particular section 100% in any state, any geographical area, then no chance of accessibility or availability of tobacco products*". Including

For peer review only - http://bmjopen.bmj.com/site/about/guidelines.xhtml

section 6b in the 'regular checklist' for the school's inspector, along with strict enforcement in and around schools was also advocated.

The need for new and achievable policies focused towards a tobacco-free generation, a shift in policy focus from only smoking to both smoking and smokeless tobacco considering the magnitude of smokeless tobacco use in India, was advocated by some interviewees. Many even suggested increasing the legal age of access to tobacco from 18 to 21 years. Vendor licensing was also identified as a key strategy to reduce tobacco accessibility. Stakeholder from the state said, *"What happens when a child goes to buy chocolate or cakes and sees lots of tobacco products also...so there is a chance he may buy tobacco products also. Because everything is sold in one shop so there is a risk of a small child getting access to tobacco shop. So here, by vendor licensing, what we are doing is, we are preventing accessibility of a child getting tobacco."*

Interviewees also spoke about developing policy or regulations to prohibit tobacco advertisement, promotion and sale on 'Over The Top' (OTT) platforms in order to protect minors from all direct and indirect methods (surrogate promotion by big tobacco) of promotion and sales on OTT platforms, which are increasingly used by young people. E-cigarettes sales on online platforms referred to the weak online compliance of the Prohibition of Electronic Cigarettes (Production, Manufacture, Import, Export, Transport, Sale, Distribution, Storage and Advertisement) Act, 2019 in the country.[21] The interviewees stressed the need of strict monitoring of regulations on OTT platforms.

All interviewees commented on successful implementation of any program being dependent on having effective and efficient inter-sectoral collaborations, from the national to grassroot levels (Panchayati Raj Institutions at village level). The active involvement of all including health, non-health, NGOs, to safeguard adolescents from tobacco was also highlighted, "*we need to wage a*

For peer review only - http://bmjopen.bmj.com/site/about/guidelines.xhtml

*battle. Finance, human resources, NGOs, policy, intersectoral collaboration not just within the health sector but with other departments also***"***.* Many interviewees suggested integrating the NTCP into other existing health and adolescent health programs like Ayushman Bharat Scheme[22] National Adolescent Health Program (Rashtriya Kishor Swasthya Karyakram)[23] and the National Programme for Prevention and Control of Cancer, Diabetes, Cardiovascular Diseases and Stroke (NPCDCS).[24]

"We have a national tobacco control programme, we have a district cancer control programme, we have oral health programme, multiple tobacco control programmes which are being implemented. We have district tobacco control programme. There are multiple overlapping issues with each of these programmes, there has to be coordinated way and mechanism in which this could be implemented at the ground level and there has to be consistency in the way in which it has to be implemented".

The significance of robust monitoring at every level was underscored by the interviewees. All tobacco-related programmes, particularly those in schools, must be monitored by the government. The school's involvement in monitoring is critical. An official from the health department said "*we conduct a programme in one school and go back to that school after 4 years, no change would be seen. So there needs for proper monitoring of every school. So, there are 3000-4000 schools in every district. Covering all schools at such large scales, can only be done through district human resources".* Similarly, many interviewees also suggested engaging civil societies, student self-help groups (like Bharat Scouts, National Cadet Corps), police functionaries for monitoring and enforcement to maximize effectiveness and increase program accessibility.

For peer review only - http://bmjopen.bmj.com/site/about/guidelines.xhtml

The need for regular evaluation of the ongoing policies and programs due to the rapid change in the tobacco industry's tactics to target adolescents and scientific advancements was also underscored. Research studies could help in identifying the loopholes or lacunae of the ongoing programs by providing evidence and encouraging them to take corrective actions to improve the programs. It was stated that there is a need to identify opportunities and weaknesses in context to regional, state and district variations and thus, a multi-pronged approach should be developed accordingly.

> *"We need to calibrate intervention, policies as per the development of industry and science and we regularly need to undertake evaluation studies to understand what are the gaps what we have done"*

DISCUSSION

The study emphasised the need for educating adolescents and enforcement agencies on tobacco use consequences, tobacco control laws and the tobacco industry's deceptive tactics targeting young people. For preventing tobacco use among adolescents, our study demonstrates the need to formulate policy with regard to tobacco-free generation, engaging adolescents meaningfully, co-creation of tobacco control adolescent interventions, strengthen and amend COTPA provisions surrounding educational institutes, improved inter-sectoral collaboration, adopting whole-societal approach, policies prioritizing smokeless tobacco control, and future research for identifying the loopholes or lacunae of the ongoing programs. The importance of having robust monitoring evaluation mechanisms were underscored to ensure most effective interventions and policies in place.

For peer review only - http://bmjopen.bmj.com/site/about/guidelines.xhtml

The introduction of COTPA[10] and 'ToFEI guidelines'[12] were considered prime steps towards tobacco control among adolescents. But even after years of implementation of these; awareness and compliance still remains a major concern, especially in rural settings and among enforcement agencies.[25–29] The study highlights the need for increasing awareness among adolescents, teachers, community members and law enforcement agencies, which is supported by previous research.[30–32] Evidence from Maharashtra (India) showcased positive effects of sensitization of police on enforcement of COTPA with increased collection of 'challans'(fines)[33] and action against shops violating section 6b of COTPA.[32]

The need to develop adolescent-driven programmes to meaningfully engage adolescents, wherein they can serve as ambassadors and be involved in creating awareness among their peers, their families, and the community about tobacco use was repeatedly emphasised in our findings. Many successful youth driven tobacco-based initiatives[34–36] have observed favourable results in terms of policy development[37], sensitization and reduced current tobacco use among youth.[38] A campaign engaging National Service Scheme (NSS) volunteers from 540 colleges in Mumbai for tobacco control resulted in improving awareness on issues of tobacco control in 176,000 students[39]; and the use of NSS volunteers[40] was also suggested in our study.

This study underscored the importance of using new media (social media) to promote tobacco control messages and restricting tobacco industry's marketing on social media, due to its widespread use among youth.[41] Existing evidence support the use of social media for tobacco control.[42–46] For example, a recent social media campaign aimed at high school students to educate them on e-cigarettes led to greater knowledge and beliefs about harmful effects of e-cigarette use, suggesting social media as a promising tool for tobacco education among young

For peer review only

people.[47] Another study also demonstrated the use of digital media to empower adolescents in smoking prevention.[48]

Our study also recognized the need to amend existing policies and development of new policies from tobacco control and prevention to 'Tobacco-Free future Generation' or 'Tobacco Endgame', to make the next generation tobacco-free. Several countries like Scotland[49] Singapore[50] New Zealand[51] Finland[52] and others are striving towards tobacco-free goal. A study weighing up the impact of New Zealand's Tobacco-Free Generation policy found that the implementation of TFG result in substantial decrease in smoking prevalence.[53] India also needs to undertake strategic steps towards a tobacco-free generation by coordinating ongoing efforts of implementing ToFEI guidelines and global youth campaigns like NMT21C (No More Tobacco in the 21st Century).[54]

India should also consider making policy changes in the legal purchase age of tobacco from 18 to 21 years as suggested by the stakeholders in our study. The evidence shows a high percentage of smokeless (75%) and smoking (77%) tobacco users in India initiate use before or up to the age of 21 years.[55] The effectiveness of this approach can be seen in California where 'Tobacco 21' was passed in 2016 and this law has led to a decrease in the illegal sale of tobacco[56,57] and a decline in cigarette smoking in high school students.[58]

India also faces the dual challenge of smoking as well as smokeless tobacco users, especially in rural areas where the prevalence of SLT users are higher,[59] in addition to most SLT users being dual users[60] (who use both SLT and smoking tobacco). Furthermore, current tobacco laws in the country are oriented towards smoking tobacco. Hence, the need for development of new policies to cater towards smokeless tobacco users was also emphasized in our study and other studies.[61] Vendor licensing as recommended in our study can help in reducing the vendor density especially near schools[62] and lower the odds of cigarette use initiation in adolescents.[63]

For peer review only - http://bmjopen.bmj.com/site/about/guidelines.xhtml

The need for extending the TAPS ban to cover OTT platforms was underscored. There is ample evidence worldwide showcasing the use (direct and indirect) of social networking sites for TAPS specifically targeting youth.[64–67] By using social media influencers, tobacco giants have found a way to circumvent policy guidelines.[68] Singapore passed laws on cyber surveillance and social media monitoring to counter the tobacco menace[69] and has also penalized and taken legal action on the TAPS violation.[70] A comprehensive ban on TAPS to ensure online streaming platforms are compliant with COTPA, is crucial for India to protect the children from exposure to tobacco products display on the OTT platforms.

Findings from our study suggest a foremost need to strengthen the school health component under the ongoing NTCP, as schools setting are deemed best for tobacco use prevention and addiction for children, school staff and families.[71,72]In terms of stricter implementation, our findings also highlight COTPA section 6b as the most important indicator for tobacco control among adolescents. In line with our findings, evidence largely supports stricter implementation of section 6b of COTPA decreased tobacco use in adolescents[73]and vice versa (increased tobacco use by adolescents to proximity and density of retail outlets near schools).[74] The TOFEI guidelines emphasized on the Self-Evaluation Scorecard for Tobacco-Free Educational Institution and this is well aligned with our study findings on regular evaluation of these guidelines.[12] With the widespread digital infrastructure in the country, compliance and monitoring of the indicators of TOFEI guidelines could benefit from the use of mobile app-based systems as suggested by stakeholders in our study. Similar app-based solutions has been implemented in few Indian states[75] and globally[76,77] and found to be impactful in improving awareness and surveillance in and around schools.

For peer review only

Our study participants emphasized that improved enforcement and implementation requires collaborative multi-sectoral partnerships, including schools, parents, officials at all levels (national, state and district), local representative, civil society and government health and non-health departments. The multi-stakeholder partnerships have been observed to be beneficial in reducing tobacco use in many parts of the world.[78–80] For instance, in New Zealand almost all educational institutes that have declared themselves 100% tobacco-free reported collaboration with external providers.[81]

Despite the novel findings presented in this paper, a few study limitations are worth considering. We have captured the perspective of various stakeholders at state, national, district and village level other than adolescents, thus the beneficiary perspective (adolescents) on tobacco control policies is missing. However, their perspective will be presented through a separate research paper carried out to understand the perception towards the current policies on tobacco control among adolescents and recommendations to address the tobacco use behaviour among them. The sampling was one of the limitations of our study as the participants for the in-depth interviews were not randomly selected, hence limiting the generalizability of the findings.

CONCLUSION

The findings of this study provide recommendations for strengthening the enforcement of existing and developing new tobacco control programmes and policies focusing on adolescents to advance India towards the ultimate goal of tobacco-free future generation and replicate such models globally.

ACKNOWLEDGEMENT

For peer review only - http://bmjopen.bmj.com/site/about/guidelines.xhtml

The authors would like to acknowledge the contributions of Dr. Nishigandha Joshi for transcribing the data from in-depth interviews. The authors thank all the interviewees who participated in this research. The paper benefited significantly from their insights and cooperation.

AUTHOR'S CONTRIBUTORS

MA, JB, SB, MB, MMK developed the research questions and study methodology. SB, MB developed an interview guide and MA, MMK, VGK provided inputs to the interview guide. The list of interviewees was developed by MA, SB, MMK. SB, MMK, RB coordinated and conducted the interviews. Data was coded and thematically analyzed by SB, MB & NT. SB, MB & NT drafted the manuscript. MA, JB, MMK, VGK, RB, IB critically reviewed the manuscript. All authors are responsible for the overall content as guarantor.

FUNDING STATEMENT

This research work is funded by the Medical Research Council of the United Kingdom under the Global Alliance for Chronic Lung diseases programme (MR/P008933/1) and was carried out as part of a collaborative project of the University of Nottingham, Manipal Academy of Higher Education, HRIDAY and the University of Bath.

COMPETING INTREST: N.A

ETHICAL APPROVAL STATEMENT:

The study was approved by the Indian Ministry of Health and Family Welfare Screening Committee (HMSC, 2017-0460), the Institutional Ethics Committee at the Centre for Chronic

For peer review only - http://bmjopen.bmj.com/site/about/guidelines.xhtml

Disease Control (CCDC_IEC_11_2018), Manipal Academy of Higher Education (MAHE EC/012/2017) and the University of Nottingham, School of Medicine Ethics Committee (OVS200317).

DATA SHARING STATEMENT

Data are available on reasonable request. As per our Institutional's data sharing policy, prior approval is required from Research Management Committee (RMC) and principal investigator (PI) of the study. After Committee's approval on the request, de-identified data can only be shared upon request.

REFERENCE

1. WHO. India loses 1% of its GDP to diseases and early deaths from tobacco use, finds WHO study [Internet]. WHO. 2021 [cited 2022 Feb 18]. Available from: https://www.who.int/india/news/detail/09-02-2021-india-loses-1-of-its-gdp-to-diseases-and-early-deaths-from-tobacco-use-finds-who-study
2. Tobacco in India [Internet]. [cited 2022 Feb 18]. Available from: https://www.who.int/india/health-topics/tobacco
3. GBD Results Tool | GHDx [Internet]. [cited 2022 Feb 18]. Available from: http://ghdx.healthdata.org/gbd-results-tool
4. International Institute for Population Sciences (IIPS) and Ministry of Health and Family Welfare. Global Adult Tobacco Survey - GATS India 2009-10 [Internet]. [cited 2022 Feb 18]. Available from: https://ntcp.nhp.gov.in/assets/document/surveys-reports-publications/Global-Adult-Tobacco-Survey-India-2009-2010-Report.pdf
5. Tata Institute of Social Sciences (TISS). Global Adult Tobacco Survey GATS 2 India 2016-17 [Internet]. TISS and Ministry of Health and Family Welfare, Government of India., New Delhi. 2017 [cited 2022 Feb 18]. Available from: https://ntcp.nhp.gov.in/assets/document/surveys-reports-publications/Global-Adult-Tobacco-Survey-Second-Round-India-2016-2017.pdf
6. Tobacco Use Among Students And Teachers Findings from the Global Youth Tobacco

For peer review only - http://bmjopen.bmj.com/site/about/guidelines.xhtml

For peer review only

Survey (GYTS) and Global School Personnel Survey (GSPS). 2003;

7. International Institute for Population Sciences (IIPS) and Ministry of Health and Family Welfare. Global Youth Tobacco Survey GYTS-4 Factsheet [Internet]. [cited 2022 Feb 18]. Available from: https://ntcp.nhp.gov.in/assets/document/National_Fact_Sheet_of_fourth_round_of_Global_Youth_Tobacco_Survey_GYTS-4.pdf

8. Chadda R, Sengupta S. Tobacco use by Indian adolescents. Tob Induc Dis [Internet]. 2002 [cited 2022 Feb 18];1(2):111. Available from: https://pubmed.ncbi.nlm.nih.gov/19570251/

9. Thakur D, Gupta A, Thakur A, Mazta SR, Sharma D. Prevalence of cigarette smoking and its predictors among school going adolescents of North India. South Asian J Cancer [Internet]. 2014 Oct 1 [cited 2022 Feb 18];3(4):193. Available from: /pmc/articles/PMC4236693/

10. Government of India. COTPA 2003 [Internet]. May 18, 2003. Available from: https://legislative.gov.in/sites/default/files/A2003-34.pdf

11. National Tobacco Control Cell Ministry of Health and Family Welfare. Operational .pdf Operational Guidelines National Tobacco Control Programme National Tobacco Control - [PDF Document] [Internet]. 2015. [cited 2022 Feb 18]. Available from: https://cupdf.com/document/operational-pdf-operational-guidelines-national-tobacco-control-programme-national.html

12. MoHFW. Guidelines for Tobacco Free Educational Institution (Revised) [Internet]. [cited 2022 Feb 18]. Available from: https://ntcp.nhp.gov.in/assets/document/TEFI-Guidelines.pdf

13. Kaur J, Jain DC. Tobacco Control Policies in India: Implementation and Challenges. Indian J Public Health [Internet]. 2011 [cited 2022 Mar 5];55(3):220. Available from: https://www.ijph.in/article.asp?issn=0019-557X;year=2011;volume=55;issue=3;spage=220;epage=227;aulast=

14. Lantz PM, Jacobson PD, Warner KE, Wasserman J, Pollack HA, Berson J, et al. Investing in youth tobacco control: a review of smoking prevention and control strategies. Tob Control [Internet]. 2000 [cited 2022 Aug 7];9(1):47–63. Available from: https://pubmed.ncbi.nlm.nih.gov/10691758/

15. Patton MQ. Qualitative evaluation and research methods, 2nd ed. Qualitative evaluation and research methods, 2nd ed. Thousand Oaks, CA, US: Sage Publications, Inc; 1990. 532 p.

16. Emmel N. Sampling and Choosing Cases in Qualitative Research: A Realist Approach [Internet]. London; 2013. Available from: https://methods.sagepub.com/book/sampling-

For peer review only

and-choosing-cases-in-qualitative-research

17. Braun V, Clarke V. Using thematic analysis in psychology. Qual Res Psychol. 2006;3(2):77–101.

18. Patton MQ. Enhancing the quality and credibility of qualitative analysis. Health Serv Res. 1999 Dec;34(5 Pt 2):1189–208.

19. Government of India. Pradhan Mantri Adarsh Gram Yojana (PMAGY) [Internet]. 2018. [cited 2022 Feb 18]. Available from: https://pmagy.gov.in/Docs/PMAGY-Guidelines-07022019-H.pdf

20. Government of India. SwachhBharat- Swachh Bharat Abhiyan a nation-wide campaign in India [Internet]. [cited 2022 Feb 18]. Available from: https://swachhbharat.mygov.in/

21. MINISTRY OF LAW AND JUSTICE. THE PROHIBITION OF ELECTRONIC CIGARETTES (PRODUCTION, MANUFACTURE, IMPORT, EXPORT, TRANSPORT, SALE, DISTRIBUTION, STORAGE AND ADVERTISEMENT) ACT, 2019 [Internet]. New Delhi; 2019 Dec [cited 2022 Feb 20]. Available from: https://ntcp.nhp.gov.in/assets/document/The-Prohibition-of-Electronic-Cigarettes-Production-Manufacture-Import-Export-Transport-Sale-Distribution-Storage-and-Advertisement)-Act-2019.pdf

22. Ministry of Health and Family Welfare G of I. Ministry of Health and Family Welfare Government of India Operational Guidelines on School Health Programme under Ayushman Bharat Health and Wellness Ambassadors partnering to build a stronger future A Joint initiative of Ministry of Health & Family Welfa. 2018;

23. Rashtriya Kishor Swasthya Karyakram (RKSK) | National Health Portal Of India [Internet]. [cited 2022 Aug 12]. Available from: https://www.nhp.gov.in/rashtriya-kishor-swasthya-karyakram-rksk_pg

24. National Programme for prevention & Control of Cancer, Diabetes, Cardiovascular Diseases & stroke (NPCDCS) :: National Health Mission [Internet]. [cited 2022 Apr 8]. Available from: https://www.nhm.gov.in/index1.php?lang=1&level=2&sublinkid=1048&lid=604

25. Chaudhary A, Thakur A, Chauhan T, Mahajan A, Barwal VK, Chamotra S, et al. Creation of a Smoke-free Environment for Children: An Assessment of Compliance to COTPA 2003 Legislation in an Urban Area. Indian Pediatr 2019 5610 [Internet]. 2019 Aug 10 [cited 2022 Mar 2];56(10):837–40. Available from: https://link.springer.com/article/10.1007/s13312-019-1609-0

26. Pradhan A, Oswal K, Padhan A, Seth S, Sarin A, Sethuraman L, et al. Cigarettes and Other Tobacco Products Act (COTPA) implementation in education institutions in India: A

For peer review only

crosssectional study. Tob Prev Cessat [Internet]. 2020 [cited 2022 Mar 2];6(September):1–7. Available from: /pmc/articles/PMC7552856/

27. Rath R, Krishnan A, Nongkynrih B, Misra P. Assessment of implementation status of Cigarettes and Other Tobacco Products Act (COTPA) and its awareness among residents in a block of Haryana. Indian J Public Health [Internet]. 2018 Apr 1 [cited 2022 Mar 2];62(2):100. Available from: https://www.ijph.in/article.asp?issn=0019-557X;year=2018;volume=62;issue=2;spage=100;epage=103;aulast=Rath

28. Goel S, Kumar R, Lal P, Singh RJ. How effective is tobacco control enforcement to protect minors: Results from subnational surveys across four districts in India. Int J Noncommunicable Dis [Internet]. 2016 [cited 2022 Mar 6];1(3):116. Available from: https://www.ijncd.org/article.asp?issn=2468-8827;year=2016;volume=1;issue=3;spage=116;epage=121;aulast=Goel

29. Ahuja N, Kathiresan J, Anand T, Isaakidis P, Bajaj D. I have heard about it for the first time from you! Implementation of tobacco control law by police personnel in India. Public Heal Action. 2019 Feb 5;8(4):194–201.

30. Rawat R, Gouda J, Shekhar C. Smokeless tobacco use among adult males in India and selected states: Assessment of education and occupation linkages. http://dx.doi.org/101080/1091135920151083507 [Internet]. 2015 Feb 17 [cited 2022 Mar 25];26(2):236–46. Available from: https://www.tandfonline.com/doi/abs/10.1080/10911359.2015.1083507

31. Doma Bhutia T, Lad N. Empowering teachers to implement tobacco free school campaigns in schools. Tob Induc Dis. 2019 Oct 12;17(1).

32. Lad N, Patil D, Pilankar A, Kadam R. Advocacy with police department for effective implementation of tobacco control polices. Tob Induc Dis. 2018 Mar 1;16(1).

33. Shinde DB. Advocacies creates champions for tobacco control through COTPA implementation & achieving sustainable development goal. Tob Induc Dis. 2021 Sep 2;19(1).

34. SWATFlorida - Students Working Against Tobacco [Internet]. [cited 2022 Mar 7]. Available from: https://www.swatflorida.com/

35. Youth Initiatives - Campaign for Tobacco-Free Kids [Internet]. [cited 2022 Feb 20]. Available from: https://www.tobaccofreekids.org/what-we-do/youth-programs

36. Youth smoking and vaping prevention [Internet]. [cited 2022 Mar 7]. Available from: https://truthinitiative.org/what-we-do/youth-smoking-prevention-education

37. Arora M, Stigler MH, Reddy KS. Effectiveness of health promotion in preventing tobacco

For peer review only

use among adolescents in India research evidence informs the national tobacco control programme in India. Glob Health Promot [Internet]. 2011 Mar 1 [cited 2022 Mar 27];18(1):09–12. Available from: https://journals.sagepub.com/doi/abs/10.1177/1757975910393163

38. Lisboa OC, Bernardes-Souza B, De Freitas Xavier LE, Almeida MR, Corrêa PCRP, Brinker TJ. A Smoking Prevention Program Delivered by Medical Students to Secondary Schools in Brazil Called "Education Against Tobacco": Randomized Controlled Trial. J Med Internet Res 2019;21(2)e12854 https//www.jmir.org/2019/2/e12854 [Internet]. 2019 Feb 21 [cited 2022 Mar 5];21(2):e12854. Available from: https://www.jmir.org/2019/2/e12854

39. Mathur A, Seth S, Sarin A. Mobilizing National Service Scheme (NSS) Volunteers for Tobacco Control in India. Tob Induc Dis. 2021 Sep 2;19(1).

40. Kumar P, Rastogi S, Sarin A. Motivating youth to mobilize community to work in tobacco control in Karnataka, India. Tob Induc Dis. 2021 Sep 2;19(1).

41. Patterns of Internet Usage Among Youths In India [Internet]. [cited 2022 Feb 20]. Available from: https://www.socialmediamatters.in/patterns-of-internet-usage-among-youths-in-india

42. Jawad M, Abass J, Hariri A, Akl EA. Social media use for public health campaigning in a low resource setting: The case of waterpipe tobacco smoking. Biomed Res Int. 2015;2015.

43. Baskerville NB, Azagba S, Norman C, McKeown K, Brown KS. Effect of a digital social media campaign on young adult smoking cessation. Nicotine Tob Res. 2016 Mar 1;18(3):351–60.

44. U.S. DEPARTMENTOF HEALTH AND HUMAN SERVICES. The Role of the Media in Promoting and Reducing Tobacco Use [Internet]. [cited 2022 Apr 8]. Available from: https://cancercontrol.cancer.gov/sites/default/files/2020-08/m19_complete.pdf

45. Luo T, Li MS, Williams D, Phillippi S, Yu Q, Kantrow S, et al. Using social media for smoking cessation interventions: a systematic review. Perspect Public Health [Internet]. 2021 Jan 1 [cited 2022 Apr 8];141(1):50–63. Available from: https://journals.sagepub.com/doi/full/10.1177/1757913920906845

46. Naslund JA, Kim SJ, Aschbrenner KA, McCulloch LJ, Brunette MF, Dallery J, et al. Systematic review of social media interventions for smoking cessation. Addict Behav. 2017 Oct 1;73:81–93.

47. Lazard AJ. Social Media Message Designs to Educate Adolescents About E-Cigarettes. J Adolesc Heal. 2021 Jan 1;68(1):130–7.

For peer review only

For peer review only - http://bmjopen.bmj.com/site/about/guidelines.xhtml

48. Park E, Chang YP. Using Digital Media to Empower Adolescents in Smoking Prevention: Mixed Methods Study. JMIR Pediatr Parent [Internet]. 2020 Jan 1 [cited 2022 Aug 2];3(1). Available from: https://pubmed.ncbi.nlm.nih.gov/32229463/

49. Scottish Government T. CREATING A TOBACCO-FREE GENERATION A Tobacco Control Strategy for Scotland CREATING A TOBACCO-FREE GENERATION A Tobacco Control Strategy for Scotland The Scottish Government, Edinburgh 2013 ii-CREATING A TOBACCO-FREE GENERATION: A TOBACCO CONTROl STRATEGy FOR SCOTlANd.

50. Khoo D, Chiam Y, Ng P, Berrick AJ, Koong HN. Phasing-out tobacco: proposal to deny access to tobacco for those born from 2000. Available from: http://tobaccocontrol.bmj.com/

51. New Zealand aims for smoke-free generation with new tobacco law | The Straits Times [Internet]. [cited 2022 Mar 7]. Available from: https://www.straitstimes.com/asia/australianz/new-zealand-aims-for-smoke-free-generation-with-new-tobacco-law

52. Tobacco-free Finland 2030 [Internet]. [cited 2022 Apr 8]. Available from: https://savutonsuomi.fi/en/

53. van der Deen FS, Wilson N, Cleghorn CL, Kvizhinadze G, Cobiac LJ, Nghiem N, et al. Impact of five tobacco endgame strategies on future smoking prevalence, population health and health system costs: two modelling studies to inform the tobacco endgame. Tob Control [Internet]. 2018 May 1 [cited 2022 Mar 7];27(3):278–86. Available from: https://tobaccocontrol.bmj.com/content/27/3/278

54. International Conference on Public Health Priorities in the 21st Century: The Endgame for Tobacco – Voice of Tobacco Victims [Internet]. [cited 2022 Mar 2]. Available from: https://vovindia.org/2013/05/23/international-conference-public-health-priorities-21st-century-endgame-tobacco/

55. Lal P, Kapoor S, Dhankhar A, Yadav A. Raising the legal age for purchase and use of tobacco to 21 years: A step towards tobacco endgame in India. Indian J Tuberc. 2021 Jan 1;68:S65–70.

56. Zhang X, Vuong TD, Andersen-Rodgers E, Roeseler A. Evaluation of California's 'Tobacco 21' law. Tob Control [Internet]. 2018 Nov 1 [cited 2022 Mar 26];27(6):656–62. Available from: https://tobaccocontrol.bmj.com/content/27/6/656

57. Ahmad S. The Cost-Effectiveness of Raising the Legal Smoking Age in California. Med Decis Mak. 2005 May;25(3):330–40.

58. CTFK. INCREASING THE MINIMUM LEGAL SALE AGE FOR TOBACCO

For peer review only - http://bmjopen.bmj.com/site/about/guidelines.xhtml

PRODUCTS TO 21 [Internet]. [cited 2022 Mar 26]. Available from: https://www.tobaccofreekids.org/assets/factsheets/0376.pdf

59. Singh PK, Yadav A, Lal P, Sinha DN, Gupta PC, Swasticharan L, et al. Dual Burden of Smoked and Smokeless Tobacco Use in India, 2009–2017: A Repeated Cross-Sectional Analysis Based on Global Adult Tobacco Survey. Nicotine Tob Res [Internet]. 2020 Dec 12 [cited 2022 Mar 26];22(12):2196–202. Available from: https://academic.oup.com/ntr/article/22/12/2196/5731397

60. Noh J-W, Kim M-H, Lee Y, Kwon YD, Kim K-B, Lee H-J, et al. Association between smokeless tobacco use and cigarette smoking amount by age. BMC Public Heal 2022 221 [Internet]. 2022 Mar 15 [cited 2022 Mar 26];22(1):1–8. Available from: https://link.springer.com/articles/10.1186/s12889-022-12929-z

61. Yadav A, Singh PK, Yadav N, Kaushik R, Chandan K, Chandra A, et al. Smokeless tobacco control in India: policy review and lessons for high-burden countries. BMJ Glob Heal [Internet]. 2020 Jul 1 [cited 2022 Mar 26];5(7):e002367. Available from: https://gh.bmj.com/content/5/7/e002367

62. Pouranik NS, Saraf S, Wright K, Pandey A, Goel S, Singh RJ, et al. Tobacco retailer density and tobacco retailers near schools in two cities of East India, Ranchi and Siliguri. Indian J Tuberc. 2021 Jan 1;68:S14–22.

63. Astor RL, Urman R, Barrington-Trimis JL, Berhane K, Steinberg J, Cousineau M, et al. Tobacco retail licensing and youth product use. Pediatrics [Internet]. 2019 Feb 1 [cited 2022 Mar 26];143(2). Available from: /pediatrics/article/143/2/e20173536/37292/Tobacco-Retail-Licensing-and-Youth-Product-Use

64. Jackler RK, Li VY, Cardiff RAL, Ramamurthi D. Promotion of tobacco products on Facebook: policy versus practice. Tob Control [Internet]. 2019 Jan 1 [cited 2022 Mar 26];28(1):67–73. Available from: https://tobaccocontrol.bmj.com/content/28/1/67

65. Freeman B, Watts C, Ayu P, Astuti S. Global tobacco advertising, promotion and sponsorship regulation: what's old, what's new and where to next? Tob Control [Internet]. 2022 Mar 1 [cited 2022 Mar 26];31(2):216–21. Available from: https://tobaccocontrol.bmj.com/content/31/2/216

66. Clendennen SL, Vandewater EA, Loukas A, Perry CL, Wilkinson A V. College students' exposure and engagement with tobacco-related social media. Tob Regul Sci. 2020 Jan 1;6(1):38–53.

67. FreemanBecky. Big tobacco uses social media to spread cancer | UICC [Internet]. UICC global cancer control. 2021 [cited 2022 Feb 20]. Available from:

For peer review only

https://www.uicc.org/blog/big-tobacco-uses-social-media-spread-cancer#

68. Mutmainnah N, Hendriyani H, Utaminingtyas I. Outsmarting regulation: how tobacco websites and social media targeting young people. Int J Commun Soc [Internet]. 2020 Jun 4 [cited 2022 Mar 26];2(1):12–9. Available from: https://pubs2.ascee.org/index.php/ijcs/article/view/108

69. Lee T. Advanced country practices in the implementation of WHO FCTC Article 13 and its guidelines Combatting cross-border advertising and depiction of tobacco in entertainment media. 2018;

70. 13 fined for illegal online sale of e-vaporisers, including one jailed for selling unregistered medicines - CNA [Internet]. [cited 2022 Feb 21]. Available from: https://www.channelnewsasia.com/singapore/vape-e-vaporisers-sale-cough-syrup-sleeping-pills-2076111

71. CTFK. HOW SCHOOLS CAN HELP STUDENTS STAY TOBACCO-FREE [Internet]. [cited 2022 Apr 8]. Available from: https://www.tobaccofreekids.org/assets/factsheets/0153.pdf

72. Adams ML, Jason LA, Pokorny S, Hunt Y. The Relationship Between School Policies and Youth Tobacco Use. J Sch Health [Internet]. 2009 Jan [cited 2022 Apr 8];79(1):17. Available from: /pmc/articles/PMC2826219/

73. Mistry R, Pednekar MS, McCarthy WJ, Resnicow K, Pimple SA, Hsieh HF, et al. Compliance with point-of-sale tobacco control policies and student tobacco use in Mumbai, India. Tob Control [Internet]. 2019 Mar 1 [cited 2022 Mar 6];28(2):220–6. Available from: https://tobaccocontrol.bmj.com/content/28/2/220

74. Henriksen L, Feighery EC, Schleicher NC, Cowling DW, Kline RS, Fortmann SP. Is adolescent smoking related to the density and proximity of tobacco outlets and retail cigarette advertising near schools? Prev Med (Baltim). 2008 Aug 1;47(2):210–4.

75. Seth S, Sarin A, Mathur A. A mobile app based solution for implementing tobacco free educational institutions (ToFEI) guidelines. Tob Induc Dis. 2021 Sep 2;19(1).

76. Pulvers K, Rice M, Stewart SL, Tong E. Tobacco Tracker: A New Tool to Support College Smoke and Tobacco Free Policies. Nicotine Tob Res [Internet]. 2022 Feb 1 [cited 2022 Mar 5];24(2):241–9. Available from: https://academic.oup.com/ntr/article/24/2/241/6398215

77. New online tool helps keep college campuses smoke and tobacco free | TRDRP [Internet]. [cited 2022 Mar 5]. Available from: https://www.trdrp.org/news/tobacco-tracker-support-healthier-campus.html

For peer review only

78. Chatterjee N, Patil D, Kadam R, Fernandes G. Tobacco-free School Policy in Maharashtra, India: A Qualitative Exploration of Implementation Facilitators and Barriers. Heal Behav Policy Rev. 2019 Feb 5;5(3):24–35.

79. Successful Collaboration Reduces Tobacco Use in Minnesota - News & Events | health.gov [Internet]. [cited 2022 Mar 7]. Available from: https://health.gov/news/201903/successful-collaboration-reduces-tobacco-use-minnesota

80. Ravindra K, Tripathy JP, Tripathy NK. Dynamics of multi-stakeholder engagement and its role in achieving high compliance of a tobacco control programme. World Dev Perspect. 2016 Sep 1;3:7–11.

81. Robertson LA, Marsh L. Smoke-free policies in New Zealand public tertiary education institutions. Health Educ Res [Internet]. 2015 Apr 1 [cited 2022 Mar 6];30(2):347–58. Available from: https://academic.oup.com/her/article/30/2/347/701723

For peer review only

Supplementary Table: An overview of qualitative data collection method, participants and themes

Themes	Stakeholders at National level	Stakeholders at state level	Stakeholders at district level	Stakeholders at village level
Existing adolescent specific tobacco control programmes to prevent tobacco uptake among adolescents	✓	✓	✓	
Existing adolescent specific tobacco control policies to prevent tobacco uptake among adolescents	✓	✓	✓	
Perception on extent of program and policy implementation	✓	✓	✓	✓
Perception on designing and implementing adolescent specific tobacco control policies	✓	✓	✓	✓
Barriers/gaps for successful implementation of adolescent specific tobacco control policies and programmes	✓	✓	✓	✓
Facilitators for successful implementation of adolescent specific tobacco control policies and programmes	✓	✓	✓	✓
Recommended strategies to prevent and reduce the use of tobacco among adolescents	✓	✓	✓	✓
Need for programme evaluation and research priorities	✓	✓	✓	

For peer review only - http://bmjopen.bmj.com/site/about/guidelines.xhtml

BMJ Publishing Group Limited (BMJ) disclaims all liability and responsibility arising from any reliance placed on this supplemental material which has been supplied by the author(s)

SRQR Reporting checklist for qualitative study.

Instructions to authors

Complete this checklist by entering the page numbers from your manuscript where readers will find each of the items listed below. SRQR reporting guidelines: O'Brien BC, Harris IB, Beckman TJ, Reed DA, Cook DA. Standards for reporting qualitative research: a synthesis of recommendations. Acad Med. 2014;89(9):1245-1251.

		Reporting Item	Page Number
**Title**			
	#1	Concise description of the nature and topic of the study identifying the study as qualitative or indicating the approach (e.g. ethnography, grounded theory) or data collection methods (e.g. interview, focus group) is recommended	Page 1
**Abstract**			Abstract
	#2	Summary of the key elements of the study using the abstract format of the intended publication; typically includes background, purpose, methods, results and conclusions	Page 3
**Introduction**			
Problem formulation	#3	Description and signifcance of the problem / phenomenon studied: review of relevant theory and empirical work; problem statement	Introduction Page 5-6
Purpose or research question	#4	Purpose of the study and specific objectives or questions	Introduction Page 6
**Methods**			
Qualitative approach and research paradigm	#5	Qualitative approach (e.g. ethnography, grounded theory, case study, phenomenolgy, narrative research) and guiding theory if appropriate; identifying the research paradigm (e.g. postpositivist, constructivist / interpretivist) is also recommended; rationale. The rationale should briefly discuss the justification for choosing that theory, approach, method or technique rather than other options available; the assumptions and limitations implicit in those choices and how those choices influence study conclusions and transferability. As appropriate the rationale for several items might be discussed together.	Recruitment and Sampling: Page 7<br>Data analysis: Page 8
Researcher characteristics and reflexivity	#6	Researchers' characteristics that may influence the research, including personal attributes, qualifications / experience, relationship with participants, assumptions and / or presuppositions; potential or actual interaction between researchers' characteristics and the research questions, approach, methods, results and / or transferability	Data collection and procedure: Page 7-8
Context	#7	Setting / site and salient contextual factors; rationale	Recruitment and Sampling: Page 7
Sampling strategy	#8	How and why research participants, documents, or events were selected; criteria for deciding when no further sampling was necessary (e.g. sampling saturation); rationale	Recruitment and sampling Page 7
Ethical issues pertaining to human subjects	#9	Documentation of approval by an appropriate ethics review board and participant consent, or explanation for lack thereof; other confidentiality and data security issues	Back Material; Page 21-22
Data collection methods	#10	Types of data collected; details of data collection procedures including (as appropriate) start and stop dates of data collection and analysis, iterative process, triangulation of sources / methods, and modification of procedures in response to evolving study findings; rationale	Data Collection and procedure Page 7
Data collection	#11	Description of instruments (e.g. interview guides,	Data collection and procedure: Page 7

For peer review only

For peer review only - http://bmjopen.bmj.com/site/about/guidelines.xhtml

instruments and technologies		questionnaires) and devices (e.g. audio recorders) used for data collection; if / how the instruments(s) changed over the course of the study	
Units of study	#12	Number and relevant characteristics of participants, documents, or events included in the study; level of participation (could be reported in results)	Results: Page 8
Data processing	#13	Methods for processing data prior to and during analysis, including transcription, data entry, data management and security, verification of data integrity, data coding, and anonymisation / deidentification of excerpts	Data analysis: Page 8
Data analysis	#14	Process by which inferences, themes, etc. were identified and developed, including the researchers involved in data analysis; usually references a specific paradigm or approach; rationale	Data analysis: Page 8
Techniques to enhance trustworthiness	#15	Techniques to enhance trustworthiness and credibility of data analysis (e.g. member checking, audit trail, triangulation); rationale	Data analysis: Page 8
**Results/findings**			
Syntheses and interpretation	#16	Main findings (e.g. interpretations, inferences, and themes); might include development of a theory or model, or integration with prior research or theory	Results: Page 9-16
Links to empirical data	#17	Evidence (e.g. quotes, field notes, text excerpts, photographs) to substantiate analytic findings	Results: Page 9-16
**Discussion**			
Intergration with prior work, implications, transferability and contribution(s) to the field	#18	Short summary of main findings; explanation of how findings and conclusions connect to, support, elaborate on, or challenge conclusions of earlier scholarship; discussion of scope of application / generalizability; identification of unique contributions(s) to scholarship in a discipline or field	Discussion: Page 16
Limitations	#19	Trustworthiness and limitations of findings	Discussion: Page 20
**Other**			
Conflicts of interest	#20	Potential sources of influence of perceived influence on study conduct and conclusions; how these were managed	Back materials Page 21
Funding	#21	Sources of funding and other support; role of funders in data collection, interpretation and reporting	Back materials Page 21

None The SRQR checklist is distributed with permission of Wolters Kluwer © 2014 by the Association of American Medical Colleges. This checklist can be completed online using https://www.goodreports.org/, a tool made by the EQUATOR Network in collaboration with Penelope.ai

For peer review only

Hennegan J, *et al. BMJ Global Health* 2020; 5:e003433. doi: 10.1136/bmjgh-2020-003433

Programme and Policy Perspective Towards a Tobacco-Free Generation: A Qualitative Study

Journal:	*BMJ Open*
Manuscript ID	bmjopen-2022-067779.R1
Article Type:	Original research
Date Submitted by the Author:	02-Jan-2023
Complete List of Authors:	Bassi, Shalini; Public Health Foundation of India, Health Promotion Division; HRIDAY,<br>Arora, Monika; HRIDAY; Public Health Foundation of India, Health Promotion Division<br>Thapliyal, Nishibha; Public Health Foundation of India, Health Promotion Division<br>Kulkarni, Muralidhar; Kasturba Medical College Manipal, Community Medicine<br>Bhagawath, Rohith; Kasturba Medical College Manipal, Community Medicine<br>Bogdanovica, Ilze; University of Nottingham, School of Medicine<br>Kamath, Veena; Kasturba Medical College Manipal, Community Medicine<br>Britton, John; University of Nottingham, Epidemiology and Public Health<br>Bains, Manpreet; University of Nottingham, School of Medicine
**Primary Subject Heading**:	Public health
Secondary Subject Heading:	Health policy, Smoking and tobacco
Keywords:	QUALITATIVE RESEARCH, PUBLIC HEALTH, Health policy < HEALTH SERVICES ADMINISTRATION & MANAGEMENT

I, the Submitting Author has the right to grant and does grant on behalf of all authors of the Work (as defined in the below author licence), an exclusive licence and/or a non-exclusive licence for contributions from authors who are: i) UK Crown employees; ii) where BMJ has agreed a CC-BY licence shall apply, and/or iii) in accordance with the terms applicable for US Federal Government officers or employees acting as part of their official duties; on a worldwide, perpetual, irrevocable, royalty-free basis to BMJ Publishing Group Ltd ("BMJ") its licensees and where the relevant Journal is co-owned by BMJ to the co-owners of the Journal, to publish the Work in this journal and any other BMJ products and to exploit all rights, as set out in our licence.

The Submitting Author accepts and understands that any supply made under these terms is made by BMJ to the Submitting Author unless you are acting as an employee on behalf of your employer or a postgraduate student of an affiliated institution which is paying any applicable article publishing charge ("APC") for Open Access articles. Where the Submitting Author wishes to make the Work available on an Open Access basis (and intends to pay the relevant APC), the terms of reuse of such Open Access shall be governed by a Creative Commons licence – details of these licences and which Creative Commons licence will apply to this Work are set out in our licence referred to above.

Other than as permitted in any relevant BMJ Author's Self Archiving Policies, I confirm this Work has not been accepted for publication elsewhere, is not being considered for publication elsewhere and does not duplicate material already published. I confirm all authors consent to publication of this Work and authorise the granting of this licence.

For peer review only

Title: Programme and Policy Perspective Towards a Tobacco-Free Generation: A Qualitative Study

Authors: Shalini Bassi[1,2]*, Monika Arora[1,2], Nishibha Thapliyal[2], Muralidhar M Kulkarni[3], Rohith Bhagawath[3], Ilze Bogdanovica[4], Veena G Kamath[3], John Britton[4], Manpreet Bains[4]

[1] Health Related Information Dissemination Amongst Youth (HRIDAY)

[2] Public Health Foundation of India (PHFI)

[3] Manipal Academy of Higher Education

[4] University of Nottingham

Corresponding Author*

Ms. Shalini Bassi,

Senior Research Scientist, Health Promotion Division, Public Health Foundation of India (PHFI), Gurgaon, Haryana, 122002, India. shalini.bassi@phfi.org

&

Consultant-HRIDAY

N-25, Second Floor, Green Park Extension, New Delhi – 110016, India. shalini@hriday-shan.org

Author Information

Ms. Shalini Bassi, Health Promotion Division, Public Health Foundation of India (PHFI), Gurugram, India. & HRIDAY, New Delhi, India. shalini.bassi@phfi.org

Dr. Monika Arora, HRIDAY, New Delhi, India & Health Promotion Division, Public Health Foundation of India (PHFI), Gurugram, Haryana, India. monika@hriday-shan.org

Dr. Nishibha Thapliyal, Health Promotion Division, Public Health Foundation of India (PHFI), Gurugram, India, nishibhathapliyal@gmail.com

For peer review only - http://bmjopen.bmj.com/site/about/guidelines.xhtml

Dr. Muralidhar M Kulkarni, Community Medicine, Kasturba Medical College Manipal, Udupi, Karnataka, India. murali.kulkarni@manipal.edu

Dr. Veena Ganesh Kamath, Community Medicine, Kasturba Medical College Manipal, Udupi, Karnataka, India. veenak@manipal.edu

Dr. Rohith Bhagawath, Community Medicine, Kasturba Medical College Manipal, Manipal Academy of Higher Education, Udupi, Karnataka, India. rohit.bhagawath@manipal.edu

Dr. Ilze Bogdanovica, School of Medicine, University of Nottingham, Nottingham, United Kingdom. Ilze.Bogdanovica@nottingham.ac.uk

Prof. John Britton, Emeritus Professor; Director, UK Centre for Tobacco & Alcohol Studies, University of Nottingham, Division of Epidemiology and Public Health, Nottingham, United Kingdom. John.Britton1@nottingham.ac.uk; j.britton@outlook.com

Dr. Manpreet Bains, School of Medicine, University of Nottingham, Nottingham, United Kingdom. manpreet.bains@nottingham.ac.uk

Word Count – Abstract- 263 words; Main document- 5140

For peer review only

ABSTRACT

Objective: This study explored multi-stakeholder perspectives on existing adolescent-specific tobacco control policies and programmes, barriers, and facilitators to their successful implementation, lessons, and recommendations for strengthening existing and developing new policies to advance India's transition towards a tobacco-free generation.

Design: Qualitative research

Setting: Semi-structured interviews were conducted with key officials at the national (India), state (Karnataka), district (Udupi), and village level. Interviews were audio-recorded, transcribed verbatim, and analysed thematically.

Participants: We conducted 38 semi-structured interviews across national (n=9), state (n=9), district (n=14), and village (n=6) levels.

Results: Our findings emphasized the need to strengthen and amend, existing provisions of the Tobacco Control Law (2003) especially in and around schools (sections 6a & 6b). Findings also suggested increasing the legal age of access to tobacco from 18 to 21 years and the development of application for compliance and monitoring indicators of ToFEI guidelines. Considering the magnitude of smokeless tobacco use in India, policies to address this problem were considered a priority. The need for strict enforcement, regular monitoring mechanisms, and impact evaluation has been stressed. Engaging adolescents meaningfully and co-creation of interventions were also emphasized. Combining both an inter-sectoral and whole-societal approach to prevent tobacco use among adolescents was also emphasized. While drafting and implementing a comprehensive national tobacco control policy the findings revealed the need to adopt a vision toward a tobacco-free generation.

For peer review only

Conclusion: Our findings highlight a need to strengthen the existing and develop new tobacco control programmes and policies focusing on adolescents. The findings revealed the need to adopt a vision toward a tobacco-free future generation and replicate such models globally.

Strengths and limitations of this study

- This study captures perspectives from a diverse range of stakeholders representing different levels of governance (national/state/district/village).
- Perspectives were captured on existing tobacco control policies and programmes, barriers, and facilitators to successful implementation and suggested recommendations for strengthening existing and developing new policies towards the ultimate goal of reaching a tobacco-free generation for India as well, as replicating such models globally.
- The beneficiary (adolescents) perspective on youth-specific tobacco control policies is missing, which will be addressed through a separate research paper.
- Our study findings are still significant and transferable despite the fact that the participants for the in-depth interviews were not randomly selected.

For peer review only

BACKGROUND

Tobacco use is a leading risk factor associated with several diseases, premature death, and disability. It is a major threat to developing and low/middle-income countries such as India, where tobacco use has reportedly led to 1.35 million annual deaths.(1,2) Furthermore, of all deaths related to non-communicable diseases in India in 2019, approximately 1.08 million deaths (17.7%) were due to tobacco use.(3) The prevalence of tobacco use among adults in India has declined from 34% to 28% between 2010 (GATS-1)(4) and 2017 (GATS-2)(5) and among school-going students aged 13-15 years from 16.9% (GYTS-1, 2003)(6) to 8.5% (GYTS-4, 2019).(7) The majority of adult tobacco users initiate use during adolescence(8) often leading to lifelong use, with low quit rates.(9) Hence, prevention of tobacco uptake among children and adolescents is of paramount importance for reducing the harms caused by tobacco use. There are many tobacco control programmes and policies, but the need for age-specific policies has gathered prominence in recent decades. Adolescent-specific policies are warranted, for instance, preventing uptake is of particular importance among them.(10)

The Government of India (GoI) has enforced various policies and programmes to prevent the uptake of tobacco by children and adolescents as a primary objective. The Section 6a of the Cigarettes and Other Tobacco Products (Prohibition of Advertisement and Regulation of Trade and Commerce, Production, Supply, and Distribution) Act (COTPA) 2003(11), stipulates the prohibition of the sale of tobacco products to and by minors; and Section 6b mandates, prohibition on the sale of tobacco products around 100 yards of educational institutions.(11) India also launched the School Programme as part of the ‘National Tobacco Control Programme’(NTCP) across public and private schools, intending to help young people to acquire the knowledge, attitudes, and skills to make informed decisions related to tobacco consumption and understand the consequences of tobacco

For peer review only - http://bmjopen.bmj.com/site/about/guidelines.xhtml

use.(12) To provide momentum to the implementation of these provisions, the Ministry of Health and Family Welfare (MoHFW)-Government of India also introduced 'Tobacco-Free Educational Institution' (ToFEI) guidelines(13), for schools, colleges, and universities, in both public and private sector, across India. These guidelines aim to generate awareness about the harmful and long-term effects of tobacco use and create a healthy and tobacco-free environment in educational institutes.

Despite several of these efforts, the implementation of tobacco control policies continues to be a challenge.(14) There is a scientific debate on whether investing in youth and adolescent smoking control and prevention efforts is effective, with mixed outcomes.(15) A growing focus is on 'anti-tobacco interest groups and their influence on strong policy implementation and success.(16) Multiple partnerships have been found beneficial in tobacco control policy implementation at various levels.(17,18) Thus, considering the perspectives of various stakeholders for formulating tobacco control policies might be the way ahead. Perspectives from multiple stakeholders increase the range and quantity of information for researchers and increase the likelihood of actions and their implementation. (19)

This paper aimed to explore the views of a diverse range of stakeholders (including policy-makers, policy-implementers, representatives from civil society, implementation agencies, and other stakeholders) on India's existing tobacco control policies and programmes focusing on adolescents, barriers to and facilitators for their successful implementation, and suggested recommendations for their successful implementation and strengthening.

For peer review only - http://bmjopen.bmj.com/site/about/guidelines.xhtml

METHODS

Design

Qualitative, one-to-one, semi-structured interviews were conducted with key officials at the national (India), state (Karnataka), district (Udupi), and village level.

Patient and Public Involvement

Patients or the public were not involved in the design, conduct, reporting, or dissemination plans of our research.

Recruitment and sampling

A purposive sampling strategy was used(19,20) where a list of 51 eligible individuals was drafted by members of the research team (MA, SB, MK), based on professional networks and level of engagement with tobacco control programmes at different levels of governance. The interviewees varied in terms of their roles, responsibilities, and seniority, to ensure that a wide range of perspectives were captured. Potential participants were approached by SB and RB via e-mail and telephone. A study information sheet and consent form were sent prior, and an interview was arranged at a time convenient for an interviewee.

Data collection and procedure

A semi-structured interview guide was developed to explore participants' views on adolescent-specific tobacco control existing policies and programmes in India, barriers to and facilitators for their successful implementation, as well as recommendations for their successful implementation and strengthening to advance India's transition towards a tobacco-free generation. The interview guide was developed by (SB, MB, with inputs from MA, MK, VK) based on the School Programme component delivered as part of NTCP(12), ToFEI guidelines by MoHFW(13), provisions of the

For peer review only - http://bmjopen.bmj.com/site/about/guidelines.xhtml

Indian Tobacco Control Act (2003)(11) specifically addressing the prevention of tobacco uptake and use by children and adolescents (Supplementary Table 1).

Interviews were conducted between July 2019 and September 2021, in English by trained qualitative researchers. National and state-level stakeholder interviews were moderated by SB, while district and village level by MK and RB. Due to the COVID-19 mitigation measures in India (e.g., travel restrictions), national and state-level interviews were conducted virtually using the Zoom platform (during December 2020-September 2021), while district and village level 'in-person' (from July 2019 -March 2020). The participants were asked to review and sign a consent form prior to interview (verbal consent was sought for virtual interviews). The consent process covered permission for data collection, audio-recording of interviews, data to be used in research publications, and dissemination whilst maintaining interviewee confidentiality and anonymity.

Data analysis

Interviews were transcribed verbatim by members of the research team. Identifiable data were anonymized and double-checked for accuracy by senior members of the research team. Transcripts were analysed by SB, NT and MB using thematic analysis(21) where both inductive and deductive approaches were adopted. To ensure the validity of interpretations(22) each individual familiarized themselves with the data separately which led to the identification of initial codes. Further readings resulted in the development of more substantive themes and sub-themes. Following this, a series of virtual calls were organized between SB, NT, and MB to discuss preliminary themes which led to a more definitive thematic framework, which was agreed upon and then applied to the remaining data (by NT) using NVivo 12.0.

For peer review only - http://bmjopen.bmj.com/site/about/guidelines.xhtml

RESULTS

In total, 38 interviews were conducted, including 9 with national-level stakeholders, 9 with state-level stakeholders (Bengaluru, Karnataka), 14 with district (Udupi) level stakeholders, and 6 village-level stakeholders. (Table 1) Interviewees included officials from the NTCP, members of the tobacco control committee (national, state, and district level), representatives from civil society organizations working in tobacco control, health, education, agriculture, food and civil supplies, police, parliamentary affairs, district administration, rural development and Panchayati raj (a system of local self-government of villages in rural India). Interviews ranged from 20 minutes to 111 minutes, with an average of 44 minutes.

Table 1: Demographic characteristics of the study participants (N=38)

	N (%)
**Sex**	
Male	26(68.4%)
Female	12 (31.6%)
**Geographical representation**	
National	9 (23.7%)
State	9 (23.7%)
District	14 (36.8%)
Village level	6 (15.8%)
**Years of working experience in tobacco control**	
0-5 years	9 (23.7%)
6-10 years	7 (18.4%)
>10 years	10 (26.3%)
Not available	12 (31.6%)
**Type of organization**	
Government	26 (68.4%)
Civil society representatives	11 (29%)
Development Agency	1 (2.6%)

Thematic analysis

For peer review only - http://bmjopen.bmj.com/site/about/guidelines.xhtml

The major themes generated were: Existing tobacco control policies and programmes focusing on adolescents in India; barriers to and facilitators for their successful implementation and recommendations for successful implementation and strengthening adolescent-specific tobacco control policies and programmes to advance India's transition towards a tobacco-free generation.

Existing tobacco control policy and programme actions focusing on adolescents in India

Our findings reported many tobacco control programmes and policies to prevent tobacco uptake among adolescents. The Government of India enacted COTPA to prevent the uptake of tobacco by children and adolescents as one of its objectives. The government has implemented several programmes as part of numerous initiatives, including school and community-based ones, to prevent adolescents from indulging in tobacco use. The interviewees believed school-based programmes or interventions to be an important way to prevent adolescents from using tobacco. Most of the interviewees spoke about the 'School Health Programme', which forms part of NTCP and a few also mentioned smaller regional or local initiatives such as, 'Yellow line campaign' (wherein, two yellow lines are painted 100 yards away from the boundary wall of an education institute demarking a 'No Tobacco Zone'); the 'Rose Campaign' (where a 'rose' flower was given to petty shop owners selling tobacco and were made aware of its consequences and asked to display boards with tobacco warnings); the 'National Population Education Project' (creating awareness or helping adolescent understand the impact of using tobacco through several activities, like poster competitions, memes, folk dance, skits, role play, mono acting, etc) and also other community-based activities with the involvement of local leaders and formation of 'Taluka level coordination committee' (a group of several villages counted together for administrative purposes) for controlling tobacco in the community.

As part of the NTCP, the participants mentioned the ongoing sensitization activities (sessions/talks/audio-visual clips) for school children, formation of anti-tobacco school committees,

For peer review only - http://bmjopen.bmj.com/site/about/guidelines.xhtml

making an anti-tobacco pledge in school assembly, organization of anti-tobacco theme-based competitions like drawing, posters, painting, quiz competitions, and relay-races. Some even spoke about anti-tobacco message competitions and role-play activities, along with annual cultural-day programmes in schools, to generate awareness of tobacco, among both students and parents. A State representative from the Education department mentioned that many school teachers are also invited to be a part of sensitization training organized by the Government which creates awareness about the ill effects of using tobacco in school children, where manuals were reportedly provided to school authorities to aid the process.

> *"We have received a manual, wherein information about cigarette content, and activities to be taken by the students to not fall into the trap is provided. There are many pictures in that, wherein it is displayed about the effects of usage of tobacco, and many more things are there in the manual. There are several activities and content that create awareness among students and we have already passed this to school authorities"*

Another component of NTCP spoken about by many interviewees was the ToFEI guidelines by the Ministry of Health and Family Welfare, a critical milestone in advancing tobacco control interventions in and around educational institutions. A national-level NGO representative highlighted that the most important indicator in ToFEI, deals with the implementation of section 6b of COTPA around the prohibition of the sale of tobacco near educational institutes:

> *"One of the great outcomes that have come out of this ToFEI programme is section 6b of COTPA which prohibits any shop selling tobacco within 100 yards of any educational institute, right? And the industry makes sure that the shops are there. One of the components*

For peer review only - http://bmjopen.bmj.com/site/about/guidelines.xhtml

in these guidelines makes us report the number of tobacco shops which are around these schools."

Barriers to the successful implementation of tobacco control programmes and policies

Most interviewees reported weak monitoring and enforcement mechanisms of NTCP and ToFEI guidelines as the primary barrier to its successful implementation. A civil society representative at the national level stated that "*there is no mechanism to monitor how many schools are tobacco-free in the state"* and another interviewee mentioned limited outreach of the guidelines "*The guidelines are not reaching upto where it has to, like if we talk about educational institutions. The Principals, the administrative authorities of these educational institutions seem to be unaware and they are unaware because nobody has reached out to them"*. Some even stated a lack of dedicated staff and budget allocation as a barrier to the programme implementation. A national-level representative said:*"There are 75 districts, there is staff only in 26 districts, 26 districts do proper reporting and monitoring but the remaining districts are not able to do because there are no staff specific to our department."*

Some mentioned the lack of sub-national level representation during the policy development process. Many stated a lack of awareness about the ongoing programmes, the tobacco control act, and its provisions among members of enforcement agencies (organizations that are responsible for programme implementation- education, police department, etc.) as a major barrier. The COVID-19 pandemic was also mentioned by some as a barrier because of the way it has overwhelmed many

For peer review only - http://bmjopen.bmj.com/site/about/guidelines.xhtml

systems, especially the health system and education due to schools being closed for more than one academic year making tobacco control trivial.

Facilitators to the successful implementation of tobacco control programmes and policies

Many proposed a bottom-up approach to facilitate the proper implementation of existing programmes and policies. Some suggested prioritizing NTCP programme at all levels, especially sub-national, along with amalgamating all school programmes under one umbrella to facilitate better implementation of NTCP. Some even mentioned implementing programmes (like NTCP and ToFEI guidelines) in coordination with other ministries, especially the police and education department for better coverage and enforcement. A national representative proposed a partnership between the ministry of health and education for the effective implementation of ToFEI guidelines. Some representatives also mentioned that tobacco control needs to be incorporated and discussed in state and district-level developmental meetings. A state-level government representative quoted, *"DLCC meeting would be a very good platform where such issues can be highlighted. Where these issues can be brought to notice of the deputy commissioner himself and when he pitches in."*

Recommendations for successful implementation and strengthening adolescent-specific tobacco control policies and programmes to advance India's transition towards a tobacco-free generation

Awareness generation and capacity building of enforcement agencies

While talking about the lessons and recommendations for strengthening tobacco control adolescent-specific efforts, the majority of the interviewees underlined the continuous need to educate adolescents about the consequences of using tobacco and how the tobacco industry uses deceptive tactics to entice the younger generation to take up their products. Using explicit examples,

For peer review only - http://bmjopen.bmj.com/site/about/guidelines.xhtml

techniques on how the tobacco industry targets young people were highlighted. More use of IEC (Information, Education, Communication) material, videos, short films, and awareness campaigns, were suggested, along with the use of newer social media platforms (*Facebook, Twitter, WhatsApp),* due to their popularity among the younger generation. Many stressed the need for targeted sensitization programmes for enforcement agencies on the importance of enforcing tobacco control laws.

Interviewees believed school-based interventions to be an important way to prevent adolescents from using tobacco. Suggesting the need for tobacco education in schools, a state representative said to "*Take up important aspects of tobacco education into the curriculum of students wherein they are taught actively that, these are habits that are detrimentally harmful, and the industry is going to try and fool them and to look for industry interference*". Some interviewees also suggested co-creating adolescent-driven projects or interventions engaging them in creating awareness among their peers, where an example from a school-based intervention in Delhi schools was referred to:

> *"The MYTRI trial, "if you see this element of peer activism in terms of interventions. So, if you do school-based interventions which are actively involving youth, as a change maker or as advocates, those interventions are more effective".*

Strengthen the enforcement and monitoring of existing tobacco control policies and programmes

A state civil society representative suggested the development of a monitoring tool by the education department, to ensure compliance with ToFEI guidelines.

> *"The educational department has to come up with a monitoring tool to assess the level of compliance with* ToFEI. *Practically someone from the education department has to physically verify the compliance. The school may upload the photographs but the physical verification by the concerned officers of the*

For peer review only - http://bmjopen.bmj.com/site/about/guidelines.xhtml

educational department and sharing the monthly progress report with the key stakeholders is more helpful to understand the level of compliance."

A national-level interviewee pointed out that the ToFEI guidelines need to be implemented properly, especially in village schools, where teachers must first be oriented, and better collaboration between ministries needs to be addressed. In rural areas, integrating ToFEI guidelines in 'Aadarsh gaon Yojana'(Model village, Rural Development Model)(23) which focuses on village development and includes social, cultural development, and social mobilization of the village community; or Clean India Mission (Swaach Bharat Abhiyan)(24) a countrywide campaign initiated by the Government of India to eliminate open defecation and improve solid waste management, was suggested.

To reduce and prevent tobacco use among youth, the majority of the interviewee emphasized establishing a comprehensive enforcement mechanism for implementing the COTPA Act in the vicinity of educational institutions. Many even stated that specific amendments to COTPA-2003, especially surrounding educational institutes concerning increasing penalties/fines were needed. The need for strengthening implementation of 6a and 6b of COTPA-2003 was highlighted by the state level Civil Society representative, "*So we have COTPA section 6a, and 6b provision. It says tobacco cannot be sold within 100 yards of any educational institution, so if you implement this particular section 100% in any state, any geographical area, then no chance of accessibility or availability of tobacco products*". Including section 6b in the 'regular checklist' for the school's inspector, along with strict enforcement in and around schools was also advocated.

The significance of robust regular monitoring at every level was underscored by the interviewees. All tobacco-related programmes, particularly those in schools, must be monitored by the government. The school's involvement in monitoring is critical. An official from the health

For peer review only - http://bmjopen.bmj.com/site/about/guidelines.xhtml

department said "*we conduct a programme in one school and go back to that school after 4 years, no change would be seen. So there need for proper monitoring of every school. So, there are 3000-4000 schools in every district. Covering all schools at such large scales can only be done through district human resources".* Similarly, many interviewees also suggested engaging civil societies, student self-help groups (like Bharat Scouts, National Cadet Corps), and police functionaries for monitoring and enforcement to maximize effectiveness and increase programme accessibility.

Development of new tobacco-free generation-centric policies

The need for new and achievable policies focused towards a tobacco-free generation, a shift in policy focus from only smoking to both smoking and smokeless tobacco considering the magnitude of smokeless tobacco use in India, was emphasized by some interviewees. Many even suggested increasing the legal age of access to tobacco from 18 to 21 years. Vendor licensing was also identified as a key strategy to reduce tobacco accessibility. A stakeholder from the state said, *"What happens when a child goes to buy chocolate or cakes and sees lots of tobacco products also...so there is a chance he may buy tobacco products also. Because everything is sold in one shop so there is a risk of a small child getting access to a tobacco shop. So here, by vendor licensing, what we are doing is, we are preventing accessibility of a child getting tobacco."*

Interviewees also spoke about developing policies or regulations to prohibit tobacco advertisement, promotion, and sale on 'Over The Top' (OTT) platforms to protect minors from all direct and indirect methods (surrogate promotion by big tobacco) of promotion and sales on OTT platforms, which are increasingly used by young people. E-cigarette sales on online platforms referred to the weak online compliance with the Prohibition of Electronic Cigarettes (Production, Manufacture, Import, Export, Transport, Sale, Distribution, Storage and Advertisement) Act, 2019 in the

For peer review only - http://bmjopen.bmj.com/site/about/guidelines.xhtml

country.(25) The interviewees stressed the need for strict monitoring of regulations on OTT platforms.

Multi-sectoral and multi-stakeholder engagement

All interviewees commented on the successful implementation of any programme being dependent on having effective and efficient inter-sectoral collaborations, from the national to grassroot levels (Panchayati Raj Institutions at the village level). The active involvement of all including health, non-health, and NGOs, to safeguard adolescents from tobacco was also highlighted, "*we need to wage a battle. Finance, human resources, NGOs, policy, intersectoral collaboration not just within the health sector but with other departments also***"**. Many interviewees suggested integrating the NTCP into other existing health and adolescent health programmes like the Ayushman Bharat Scheme(26) National Adolescent Health Programme (Rashtriya Kishor Swasthya Karyakram)(27) and the National Programme for Prevention and Control of Cancer, Diabetes, Cardiovascular Diseases and Stroke (NPCDCS).(28)

"We have a national tobacco control programme, we have a district cancer control programme, we have oral health programme, multiple tobacco control programmes which are being implemented. We have district tobacco control programme. There are multiple overlapping issues with each of these programmes, there has to be coordinated way and mechanism in which this could be implemented at the ground level and there has to be consistency in the way in which it has to be implemented".

Regular impact evaluation

The need for regular evaluation of the ongoing policies and programmes due to the rapid change in the tobacco industry's tactics to target adolescents and scientific advancements was also underscored. Research studies could help in identifying the loopholes or lacunae of the ongoing

For peer review only - http://bmjopen.bmj.com/site/about/guidelines.xhtml

programmes by providing evidence and encouraging them to take corrective actions to improve the programmes. It was stated that there is a need to identify opportunities and weaknesses in the context of regional, state, and district variations, and thus, a multi-pronged approach should be developed accordingly.

"We need to calibrate intervention, and policies as per the development of industry and science and we regularly need to undertake impact evaluation studies to understand what are the gaps what we have done"

DISCUSSION

The study emphasised the need for educating adolescents and enforcement agencies on tobacco use consequences, tobacco control laws, and the tobacco industry's deceptive tactics targeting young people. For preventing tobacco use among adolescents, our study demonstrates the need to formulate policy concerning tobacco-free generation, engaging adolescents meaningfully, co-creation of tobacco control adolescent interventions, strengthening and amending COTPA provisions surrounding educational institutes, improving multi-sectoral and multi-stakeholder engagement, adopting the whole-societal approach, policies prioritizing smokeless tobacco control, and future research for identifying the loopholes or lacunae of the ongoing programmes. The importance of having robust monitoring and evaluation mechanisms was underscored to ensure the most effective interventions and policies in place.

The introduction of COTPA(11) and 'ToFEI guidelines'(13) were considered prime steps towards tobacco control among adolescents. But even after years of implementation of these; awareness and compliance remain a major concern, especially in rural settings and among enforcement agencies.(29–33) The study highlights the need for increasing awareness among adolescents,

For peer review only - http://bmjopen.bmj.com/site/about/guidelines.xhtml

teachers, community members, and law enforcement agencies, which is supported by previous research.(34–36) Evidence from Maharashtra (India) showcased the positive effects of sensitization of police on enforcement of COTPA with an increased collection of 'challans'(fines)(37) and action against shops violating section 6b of COTPA.(36)

The need to develop adolescent-driven programmes to meaningfully engage adolescents, wherein they can serve as ambassadors and be involved in creating awareness among their peers, their families, and the community about tobacco use was repeatedly emphasised in our findings. Many successful youth driven tobacco-based initiatives(38–40) have observed favourable results in terms of policy development(41), sensitization and reduced current tobacco use among youth.(42) A campaign engaging National Service Scheme (NSS) volunteers from 540 colleges in Mumbai for tobacco control resulted in improving awareness of tobacco control issues in 176,000 students(43), and the use of NSS volunteers(44) was also suggested in our study.

This study underscored the importance of using new media (social media) to promote tobacco control messages and restricting tobacco industry's marketing on social media, due to its widespread use among youth.(45) Existing evidence supports the use of social media for tobacco control.(46–50) For example, a recent social media campaign aimed at high school students to educate them on e-cigarettes led to greater knowledge and beliefs about the harmful effects of e-cigarette use, suggesting social media as a promising tool for tobacco education among young people.(51) Another study also demonstrated the use of digital media to empower adolescents in smoking prevention.(52)

Our study also recognized the need to amend existing policies and development of new policies from tobacco control and prevention to 'Tobacco-Free future Generation' or 'Tobacco Endgame', to make the next generation tobacco-free. Several countries like Scotland(53) Singapore(54) New

For peer review only - http://bmjopen.bmj.com/site/about/guidelines.xhtml

Zealand(55) Finland(56) and others are striving towards a tobacco-free goal. A study weighing up the impact of New Zealand's Tobacco-Free Generation policy found that the implementation of TFG results in a substantial decrease in smoking prevalence.(57) India also needs to undertake strategic steps towards a tobacco-free generation by coordinating ongoing efforts of implementing ToFEI guidelines and global youth campaigns like NMT21C (No More Tobacco in the 21st Century).(58)

India should also consider making policy changes in the legal purchase age of tobacco from 18 to 21 years as suggested by the stakeholders in our study. The evidence shows a high percentage of smokeless (75%) and smoking (77%) tobacco users in India initiate use before or up to the age of 21 years.(59) This is also supported by the scientific evidence that the brain continues to develop until about the age of 25 years. During this age range, brain growth is not complete and susceptibility to the damaging effects of tobacco smoke may be enhanced.(60,61) The effectiveness of this approach can be seen in California where 'Tobacco 21' was passed in 2016 and this law has led to a decrease in the illegal sale of tobacco(62,63) and a decline in cigarette smoking in high school students.(64)

India also faces the dual challenge of smoking as well as smokeless tobacco users, especially in rural areas where the prevalence of SLT users is higher,(65) in addition to most SLT users being dual users(66) (who use both SLT and smoking tobacco). Furthermore, current tobacco laws in the country are oriented toward smoking tobacco. Hence, the need for the development of new policies to cater to smokeless tobacco users was also emphasized in our study and other studies.(67) Vendor licensing as recommended in our study can help in reducing the vendor density, especially near schools(68), and lower the odds of cigarette use initiation in adolescents.(69)

For peer review only - http://bmjopen.bmj.com/site/about/guidelines.xhtml

The need for extending the TAPS ban to cover OTT platforms was underscored. There is ample evidence worldwide showcasing the use (direct and indirect) of social networking sites for TAPS specifically targeting youth.(70–73) By using social media influencers, tobacco giants have found a way to circumvent policy guidelines.(74) Singapore passed laws on cyber surveillance and social media monitoring to counter the tobacco menace(75) and has also penalized and taken legal action on the TAPS violation.(76) A comprehensive ban on TAPS to ensure online streaming platforms are compliant with COTPA, is crucial for India to protect children from exposure to tobacco products displayed on the OTT platforms.

Findings from our study suggest a foremost need to strengthen the school health component under the ongoing NTCP, as school settings are deemed best for tobacco use prevention and addiction for children, school staff, and families. Families have an enormous influence on a child's tobacco use behaviour(77,78) and this has also been reported in our study. Thus, parents, particularly in villages need to be sensitized and should be involved in school tobacco-free efforts to reduce tobacco exposure at home. In terms of stricter implementation, our findings also highlight COTPA section 6b as the most important indicator for tobacco control among adolescents. In line with our findings, evidence largely supports the stricter implementation of section 6b of COTPA decreased tobacco use in adolescents(79) and vice versa (increased tobacco use by adolescents to proximity and density of retail outlets near schools).(80) The ToFEI guidelines emphasized the Self-Evaluation Scorecard for Tobacco-Free Educational Institutions and this is well aligned with our study findings on the regular evaluation of these guidelines.(13) With the widespread digital infrastructure in the country, compliance, and monitoring of the indicators of ToFEI guidelines could benefit from the use of mobile app-based systems as suggested by stakeholders in our study. Similar app-based solutions

For peer review only - http://bmjopen.bmj.com/site/about/guidelines.xhtml

has been implemented in few Indian states(81) and globally(82,83) and found to be impactful in improving awareness and surveillance in and around schools.

Our study participants emphasized that improved enforcement and implementation require collaborative multi-sectoral partnerships, including schools, parents, officials at all levels (national, state and district), local representatives, civil society, and government health and non-health departments. The multi-stakeholder partnerships have been observed to be beneficial in reducing tobacco use in many parts of the world.(17,84,85) For instance, in New Zealand almost all educational institutes that have declared themselves 100% tobacco-free reported collaboration with external providers.(86)

Despite the novel findings presented in this paper, a few study limitations are worth considering. We have captured the perspectives from a diverse range of stakeholders representing a different level of governance (at national, state, district, and village level) other than adolescents, thus the beneficiary perspective (adolescents) on tobacco control policies is missing. However, their perspective will be presented through a separate research paper carried out to understand the perception towards the current policies on tobacco control among adolescents and recommendations to address the tobacco use behaviour among them. The sampling was one of the limitations of our study as the participants for the in-depth interviews were purposely selected. Nevertheless, we had a fairly balanced response in terms of stakeholders from different levels of governance and years of experience in tobacco control making our study findings transferable and significant. Additionally, the lack of comparison of study findings to a framework can be looked at as a limitation that can be explored in future research.

CONCLUSION

For peer review only - http://bmjopen.bmj.com/site/about/guidelines.xhtml

The findings of this study provide recommendations for strengthening the enforcement of existing and developing new tobacco control programmes and policies focusing on adolescents to advance India towards the ultimate goal of a tobacco-free future generation and replicate such models globally.

ACKNOWLEDGEMENT

The authors would like to acknowledge the contributions of Dr. Nishigandha Joshi for transcribing the data from in-depth interviews. The authors thank all the interviewees who participated in this research. The paper benefited significantly from their insights and cooperation.

AUTHOR'S CONTRIBUTORS

MA, JB, SB, MB, MMK developed the research questions and study methodology. SB, MB developed an interview guide and MA, MMK, VGK provided inputs to the interview guide. The list of interviewees was developed by MA, SB, MMK. SB, MMK, RB coordinated and conducted the interviews. Data was coded and thematically analysed by SB, MB & NT. SB, MB & NT drafted the manuscript. MA, JB, MMK, VGK, RB, IB critically reviewed the manuscript. All authors are responsible for the overall content as guarantor.

FUNDING STATEMENT

This research work is funded by the Medical Research Council of the United Kingdom under the Global Alliance for Chronic Lung diseases programme (MR/P008933/1) and was carried out as part of a collaborative project of the University of Nottingham, Manipal Academy of Higher Education, HRIDAY and the University of Bath.

For peer review only - http://bmjopen.bmj.com/site/about/guidelines.xhtml

COMPETING INTREST: N.A

ETHICAL APPROVAL STATEMENT:

The study was approved by the Indian Ministry of Health and Family Welfare Screening Committee (HMSC, 2017-0460), the Institutional Ethics Committee at the Centre for Chronic Disease Control (CCDC_IEC_11_2018), Manipal Academy of Higher Education (MAHE EC/012/2017) and the University of Nottingham, School of Medicine Ethics Committee (OVS200317).

DATA SHARING STATEMENT

Data are available on reasonable request. As per our Institutional data sharing policy, prior approval is required from the Research Management Committee (RMC) and the principal investigator (PI) of the study. After Committee's approval of the request, de-identified data can only be shared upon request.

REFERENCES

1. WHO. India loses 1% of its GDP to diseases and early deaths from tobacco use, finds WHO study [Internet]. WHO. 2021. Available from: https://www.who.int/india/news/detail/09-02-2021-india-loses-1-of-its-gdp-to-diseases-and-early-deaths-from-tobacco-use-finds-who-study[Accessed 18 Feb 2022]
2. Tobacco in India [Internet]. [cited 2022 Feb 18]. Available from: https://www.who.int/india/health-topics/tobacco
3. GBD Results Tool | GHDx [Internet]. Available from: http://ghdx.healthdata.org/gbd-results-tool. [Accessed 18 Feb 2022]
4. International Institute for Population Sciences (IIPS) and Ministry of Health and Family Welfare. Global Adult Tobacco Survey - GATS India 2009-10 [Internet]. Available from: https://ntcp.nhp.gov.in/assets/document/surveys-reports-publications/Global-Adult-Tobacco-Survey-India-2009-2010-Report.pdf. [Accessed 18 Feb 2022]
5. Tata Institute of Social Sciences (TISS). Global Adult Tobacco Survey GATS 2 India 2016-17 [Internet]. TISS and Ministry of Health and Family Welfare, Government of India., New Delhi. 201. Available from: https://ntcp.nhp.gov.in/assets/document/surveys-reports-publications/Global-Adult-Tobacco-Survey-Second-Round-India-2016-2017.pdf [Accessed 18 Feb 2022]

For peer review only - http://bmjopen.bmj.com/site/about/guidelines.xhtml

6. Tobacco Use Among Students And Teachers Findings from the Global Youth Tobacco Survey (GYTS) and Global School Personnel Survey (GSPS). 2003;
7. International Institute for Population Sciences (IIPS) and Ministry of Health and Family Welfare. Global Youth Tobacco Survey GYTS-4 Factsheet [Internet]. Available from: https://ntcp.nhp.gov.in/assets/document/National_Fact_Sheet_of_fourth_round_of_Global_Youth_Tobacco_Survey_GYTS-4.pdf [Accessed 18 Feb 2022]
8. Chadda R, Sengupta S. Tobacco use by Indian adolescents. Tob Induc Dis. 2002;1(2):111. Available from: https://pubmed.ncbi.nlm.nih.gov/19570251/
9. Thakur D, Gupta A, Thakur A, Mazta SR, Sharma D. Prevalence of cigarette smoking and its predictors among school going adolescents of North India. South Asian J Cancer. 2014;3(4):193. Available from: /pmc/articles/PMC4236693/
10. Ranabhat CL, Kim CB, Park MB, Jakovljevic M. Situation, impacts, and future challenges of tobacco control policies for youth: An explorative systematic policy review. Front Pharmacol. 2019;10:981.
11. Government of India. COTPA 2003 [Internet]. May 18, 2003. Available from: https://legislative.gov.in/sites/default/files/A2003-34.pdf
12. National Tobacco Control Cell Ministry of Health and Family Welfare. Operational .pdf Operational Guidelines National Tobacco Control Programme National Tobacco Control - [PDF Document] [Internet]. 2015. Available from: https://cupdf.com/document/operational-pdf-operational-guidelines-national-tobacco-control-programme-national.html [Accessed 18 Feb 2022]
13. MoHFW. Guidelines for Tobacco Free Educational Institution (Revised) [Internet]. Available from: https://ntcp.nhp.gov.in/assets/document/TEFI-Guidelines.pdf [Accessed 18 Feb 2022]
14. Kaur J, Jain DC. Tobacco Control Policies in India: Implementation and Challenges. Indian J Public Health. 2011;55(3):220. Available from: https://www.ijph.in/article.asp?issn=0019-557X;year=2011;volume=55;issue=3;spage=220;epage=227;aulast=
15. Lantz PM, Jacobson PD, Warner KE, Wasserman J, Pollack HA, Berson J, et al. Investing in youth tobacco control: a review of smoking prevention and control strategies. Tob Control. 2000;9(1):47–63.
16. Kuijpers TG, Kunst AE, Willemsen MC. Who calls the shots in tobacco control policy? Policy monopolies of pro and anti-tobacco interest groups across six European countries. BMC Public Health. 2019;19(1):1–13. Available from: https://bmcpublichealth.biomedcentral.com/articles/10.1186/s12889-019-7158-6
17. Ravindra K, Tripathy JP, Tripathy NK. Dynamics of multi-stakeholder engagement and its role in achieving high compliance of a tobacco control programme. World Dev Perspect. 2016 Sep 1;3:7–11.
18. MacGregor S. Editorial: Ireland's workplace smoking ban. http://dx.doi.org/101080/09687630412331330074 [Internet]. 2009;12(SUPPL. 1):1–136. Available from: https://www.tandfonline.com/doi/abs/10.1080/09687630412331330074
19. Patton MQ. Qualitative evaluation and research methods, 2nd ed. Qualitative evaluation and research methods, 2nd ed. Thousand Oaks, CA, US: Sage Publications, Inc; 1990. 532 p.
20. Emmel N. Sampling and Choosing Cases in Qualitative Research: A Realist Approach [Internet]. London; 2013. Available from: https://methods.sagepub.com/book/sampling-and-choosing-cases-in-qualitative-research

For peer review only - http://bmjopen.bmj.com/site/about/guidelines.xhtml

21. Braun V, Clarke V. Using thematic analysis in psychology. Qual Res Psychol. 2006;3(2):77–101.
22. Patton MQ. Enhancing the quality and credibility of qualitative analysis. Health Serv Res. 1999 Dec;34(5 Pt 2):1189–208.
23. Government of India. Pradhan Mantri Adarsh Gram Yojana (PMAGY) [Internet]. 2018. Available from: https://pmagy.gov.in/Docs/PMAGY-Guidelines-07022019-H.pdf[Accessed 18 Feb 2022]
24. Government of India. SwachhBharat- Swachh Bharat Abhiyan a nation-wide campaign in India [Internet]. Available from: https://swachhbharat.mygov.in/[Accessed 18 Feb 2022]
25. MINISTRY OF LAW AND JUSTICE. THE PROHIBITION OF ELECTRONIC CIGARETTES (PRODUCTION, MANUFACTURE, IMPORT, EXPORT, TRANSPORT, SALE, DISTRIBUTION, STORAGE AND ADVERTISEMENT) ACT, 2019 [Internet]. New Delhi; 2019 Dec. Available from: https://ntcp.nhp.gov.in/assets/document/The-Prohibition-of-Electronic-Cigarettes-Production-Manufacture-Import-Export-Transport-Sale-Distribution-Storage-and-Advertisement)-Act-2019.pdf [Accessed 18 Feb 2022]
26. Ministry of Health and Family Welfare G of I. Ministry of Health and Family Welfare Government of India Operational Guidelines on School Health Programme under Ayushman Bharat Health and Wellness Ambassadors partnering to build a stronger future A Joint initiative of Ministry of Health & Family Welfa. 2018;
27. Rashtriya Kishor Swasthya Karyakram (RKSK) | National Health Portal Of India [Internet]. [cited 2022 Aug 12]. Available from: https://www.nhp.gov.in/rashtriya-kishor-swasthya-karyakram-rksk_pg
28. National Programme for prevention & Control of Cancer, Diabetes, Cardiovascular Diseases & stroke (NPCDCS) :: National Health Mission [Internet]. Available from: https://www.nhm.gov.in/index1.php?lang=1&level=2&sublinkid=1048&lid=604[Accessed 8 Apr 2022]
29. Chaudhary A, Thakur A, Chauhan T, Mahajan A, Barwal VK, Chamotra S, et al. Creation of a Smoke-free Environment for Children: An Assessment of Compliance to COTPA 2003 Legislation in an Urban Area. Indian Pediatr 2019 5610 [Internet]. 2019;56(10):837–40. Available from: https://link.springer.com/article/10.1007/s13312-019-1609-0
30. Pradhan A, Oswal K, Padhan A, Seth S, Sarin A, Sethuraman L, et al. Cigarettes and Other Tobacco Products Act (COTPA) implementation in education institutions in India: A crosssectional study. Tob Prev Cessat [Internet]. 202;6(September):1–7. Available from: /pmc/articles/PMC7552856/
31. Rath R, Krishnan A, Nongkynrih B, Misra P. Assessment of implementation status of Cigarettes and Other Tobacco Products Act (COTPA) and its awareness among residents in a block of Haryana. Indian J Public Health. 2018;62(2):100. Available from: https://www.ijph.in/article.asp?issn=0019-557X;year=2018;volume=62;issue=2;spage=100;epage=103;aulast=Rath
32. Goel S, Kumar R, Lal P, Singh RJ. How effective is tobacco control enforcement to protect minors: Results from subnational surveys across four districts in India. Int J Noncommunicable Dis. 2016;1(3):116. Available from: https://www.ijncd.org/article.asp?issn=2468-8827;year=2016;volume=1;issue=3;spage=116;epage=121;aulast=Goel
33. Ahuja N, Kathiresan J, Anand T, Isaakidis P, Bajaj D. I have heard about it for the first time from you! Implementation of tobacco control law by police personnel in India. Public Heal Action. 2019 Feb 5;8(4):194–201.

For peer review only - http://bmjopen.bmj.com/site/about/guidelines.xhtml

34. Rawat R, Gouda J, Shekhar C. Smokeless tobacco use among adult males in India and selected states: Assessment of education and occupation linkages.. 2015;26(2):236–46. http://dx.doi.org/101080/10911359201510835O7
35. Doma Bhutia T, Lad N. Empowering teachers to implement tobacco free school campaigns in schools. Tob Induc Dis. 2019 Oct 12;17(1).
36. Lad N, Patil D, Pilankar A, Kadam R. Advocacy with police department for effective implementation of tobacco control polices. Tob Induc Dis. 2018 Mar 1;16(1).
37. Shinde DB. Advocacies creates champions for tobacco control through COTPA implementation & achieving sustainable development goal. Tob Induc Dis. 2021 Sep 2;19(1).
38. SWATFlorida - Students Working Against Tobacco [Internet]. Available from: https://www.swatflorida.com/[Accessed 7 Mar 2022]
39. Youth Initiatives - Campaign for Tobacco-Free Kids [Internet]. Available from: https://www.tobaccofreekids.org/what-we-do/youth-programs [Accessed 7 Mar 2022]
40. Youth smoking and vaping prevention [Internet]. [cited 2022 Mar 7]. Available from: https://truthinitiative.org/what-we-do/youth-smoking-prevention-education
41. Arora M, Stigler MH, Reddy KS. Effectiveness of health promotion in preventing tobacco use among adolescents in India research evidence informs the national tobacco control programme in India. Glob Health Promot. 2011;18(1):09–12. Available from: https://journals.sagepub.com/doi/abs/10.1177/1757975910393163
42. Lisboa OC, Bernardes-Souza B, De Freitas Xavier LE, Almeida MR, Corrêa PCRP, Brinker TJ. A Smoking Prevention Program Delivered by Medical Students to Secondary Schools in Brazil Called "Education Against Tobacco": Randomized Controlled Trial. J Med Internet Res 2019;21(2)e12854
43. Mathur A, Seth S, Sarin A. Mobilizing National Service Scheme (NSS) Volunteers for Tobacco Control in India. Tob Induc Dis. 2021 Sep 2;19(1).
44. Kumar P, Rastogi S, Sarin A. Motivating youth to mobilize community to work in tobacco control in Karnataka, India. Tob Induc Dis. 2021 Sep 2;19(1).
45. Patterns of Internet Usage Among Youths In India [Internet]. Available from: https://www.socialmediamatters.in/patterns-of-internet-usage-among-youths-in-india[Accessed 20 Feb 2022]
46. Jawad M, Abass J, Hariri A, Akl EA. Social media use for public health campaigning in a low resource setting: The case of waterpipe tobacco smoking. Biomed Res Int. 2015;2015.
47. Baskerville NB, Azagba S, Norman C, McKeown K, Brown KS. Effect of a digital social media campaign on young adult smoking cessation. Nicotine Tob Res. 2016 Mar 1;18(3):351–60.
48. U.S. DEPARTMENTOF HEALTH AND HUMAN SERVICES. The Role of the Media in Promoting and Reducing Tobacco Use [Internet]. Available from: https://cancercontrol.cancer.gov/sites/default/files/2020-08/m19_complete.pdf[Accessed 8 Apr 2022]
49. Luo T, Li MS, Williams D, Phillippi S, Yu Q, Kantrow S, et al. Using social media for smoking cessation interventions: a systematic review. Perspect Public Health [Internet]. 2021 Jan 1 [cited 2022 Apr 8];141(1):50–63. Available from: https://journals.sagepub.com/doi/full/10.1177/1757913920906845
50. Naslund JA, Kim SJ, Aschbrenner KA, McCulloch LJ, Brunette MF, Dallery J, et al. Systematic review of social media interventions for smoking cessation. Addict Behav. 2017 Oct 1;73:81–93.

For peer review only - http://bmjopen.bmj.com/site/about/guidelines.xhtml

51. Lazard AJ. Social Media Message Designs to Educate Adolescents About E-Cigarettes. J Adolesc Heal. 2021 Jan 1;68(1):130–7.
52. Park E, Chang YP. Using Digital Media to Empower Adolescents in Smoking Prevention: Mixed Methods Study. JMIR Pediatr Parent. 2020;3(1). Available from: https://pubmed.ncbi.nlm.nih.gov/32229463/
53. Scottish Government T. CREATING A TOBACCO-FREE GENERATION A Tobacco Control Strategy for Scotland CREATING A TOBACCO-FREE GENERATION A Tobacco Control Strategy for Scotland The Scottish Government, Edinburgh 2013 ii-CREATING A TOBACCO-FREE GENERATION: A TOBACCO CONTROl STRATEGy FOR SCOTlANd.
54. Khoo D, Chiam Y, Ng P, Berrick AJ, Koong HN. Phasing-out tobacco: proposal to deny access to tobacco for those born from 2000. Available from: http://tobaccocontrol.bmj.com/
55. New Zealand aims for smoke-free generation with new tobacco law | The Straits Times [Internet]. Available from: https://www.straitstimes.com/asia/australianz/new-zealand-aims-for-smoke-free-generation-with-new-tobacco-law[Accessed 7 Mar 2022]
56. Tobacco-free Finland 2030 [Internet]. Available from: https://savutonsuomi.fi/en/[Accessed 8 Apr 2022]
57. van der Deen FS, Wilson N, Cleghorn CL, Kvizhinadze G, Cobiac LJ, Nghiem N, et al. Impact of five tobacco endgame strategies on future smoking prevalence, population health and health system costs: two modelling studies to inform the tobacco endgame. Tob Control. 2018;27(3):278–86. Available from: https://tobaccocontrol.bmj.com/content/27/3/278
58. International Conference on Public Health Priorities in the 21st Century: The Endgame for Tobacco – Voice of Tobacco Victims [Internet]. Available from: https://vovindia.org/2013/05/23/international-conference-public-health-priorities-21st-century-endgame-tobacco/[Accessed 2 Mar 2022]
59. Lal P, Kapoor S, Dhankhar A, Yadav A. Raising the legal age for purchase and use of tobacco to 21 years: A step towards tobacco endgame in India. Indian J Tuberc. 2021 Jan 1;68:S65–70.
60. Bonnie RJ, Stratton K, Kwan LY, Products C on the PHI of R the MA for PT, Practice B on PH and PH, Medicine I of. Public Health Implications of Raising the Minimum Age of Legal Access to Tobacco Products [Internet]. Public Health Implications of Raising the Minimum Age of Legal Access to Tobacco Products. National Academies Press (US); 2015. 1–378 p. Available from: https://www.ncbi.nlm.nih.gov/books/NBK310412/[Accessed 30 Dec 2022]
61. Understanding the Teen Brain - Health Encyclopedia - University of Rochester Medical Center [Internet]. Available from: https://www.urmc.rochester.edu/encyclopedia/content.aspx?ContentTypeID=1&ContentID=3051 [Accessed 30 Dec 2022]
62. Zhang X, Vuong TD, Andersen-Rodgers E, Roeseler A. Evaluation of California's 'Tobacco 21' law. Tob Control. 2018;27(6):656–62.
63. Ahmad S. The Cost-Effectiveness of Raising the Legal Smoking Age in California. Med Decis Mak. 2005 May;25(3):330–40.
64. CTFK. INCREASING THE MINIMUM LEGAL SALE AGE FOR TOBACCO PRODUCTS TO 21 [Internet]. Available from: https://www.tobaccofreekids.org/assets/factsheets/0376.pdf [Accessed 26 Mar 2022]
65. Singh PK, Yadav A, Lal P, Sinha DN, Gupta PC, Swasticharan L, et al. Dual Burden of

For peer review only

For peer review only - http://bmjopen.bmj.com/site/about/guidelines.xhtml

Smoked and Smokeless Tobacco Use in India, 2009–2017: A Repeated Cross-Sectional Analysis Based on Global Adult Tobacco Survey. Nicotine Tob Res. 2020;22(12):2196–202.
66. Noh J-W, Kim M-H, Lee Y, Kwon YD, Kim K-B, Lee H-J, et al. Association between smokeless tobacco use and cigarette smoking amount by age. BMC Public Heal. 2022;22(1):1–8. Available from: https://link.springer.com/articles/10.1186/s12889-022-12929-z
67. Yadav A, Singh PK, Yadav N, Kaushik R, Chandan K, Chandra A, et al. Smokeless tobacco control in India: policy review and lessons for high-burden countries. BMJ Glob Heal. 2020;5(7):e002367. Available from: https://gh.bmj.com/content/5/7/e002367
68. Pouranik NS, Saraf S, Wright K, Pandey A, Goel S, Singh RJ, et al. Tobacco retailer density and tobacco retailers near schools in two cities of East India, Ranchi and Siliguri. Indian J Tuberc. 2021 Jan 1;68:S14–22.
69. Astor RL, Urman R, Barrington-Trimis JL, Berhane K, Steinberg J, Cousineau M, et al. Tobacco retail licensing and youth product use. Pediatrics. 2019;143(2). Available from: /pediatrics/article/143/2/e20173536/37292/Tobacco-Retail-Licensing-and-Youth-Product-Use
70. Jackler RK, Li VY, Cardiff RAL, Ramamurthi D. Promotion of tobacco products on Facebook: policy versus practice. Tob Control. 2019;28(1):67–73. Available from: https://tobaccocontrol.bmj.com/content/28/1/67
71. Freeman B, Watts C, Ayu P, Astuti S. Global tobacco advertising, promotion and sponsorship regulation: what's old, what's new and where to next? Tob Control. 2022;31(2):216–21.
72. Clendennen SL, Vandewater EA, Loukas A, Perry CL, Wilkinson A V. College students' exposure and engagement with tobacco-related social media. Tob Regul Sci. 2020 Jan 1;6(1):38–53.
73. FreemanBecky. Big tobacco uses social media to spread cancer | UICC [Internet]. UICC global cancer control. 2021. Available from: https://www.uicc.org/blog/big-tobacco-uses-social-media-spread-cancer# [Accessed 20 Feb 2022]
74. Mutmainnah N, Hendriyani H, Utaminingtyas I. Outsmarting regulation: how tobacco websites and social media targeting young people. Int J Commun Soc. 2020;2(1):12–9. Available from: https://pubs2.ascee.org/index.php/ijcs/article/view/108
75. Lee T. Advanced country practices in the implementation of WHO FCTC Article 13 and its guidelines Combatting cross-border advertising and depiction of tobacco in entertainment media. 2018;
76. 13 fined for illegal online sale of e-vaporisers, including one jailed for selling unregistered medicines - CNA [Internet]. Available from: https://www.channelnewsasia.com/singapore/vape-e-vaporisers-sale-cough-syrup-sleeping-pills-2076111 [Accessed 21 Feb 2022]
77. CTFK. HOW SCHOOLS CAN HELP STUDENTS STAY TOBACCO-FREE [Internet]. Available from: https://www.tobaccofreekids.org/assets/factsheets/0153.pdf[Accessed 8 Apr 2022]
78. Adams ML, Jason LA, Pokorny S, Hunt Y. The Relationship Between School Policies and Youth Tobacco Use. J Sch Health. 2009;79(1):17. Available from: /pmc/articles/PMC2826219/
79. Mistry R, Pednekar MS, McCarthy WJ, Resnicow K, Pimple SA, Hsieh HF, et al. Compliance with point-of-sale tobacco control policies and student tobacco use in Mumbai,

For peer review only - http://bmjopen.bmj.com/site/about/guidelines.xhtml

India. Tob Control. 2019;28(2):220–6.
80. Henriksen L, Feighery EC, Schleicher NC, Cowling DW, Kline RS, Fortmann SP. Is adolescent smoking related to the density and proximity of tobacco outlets and retail cigarette advertising near schools? Prev Med (Baltim). 2008;47(2):210–4.
81. Seth S, Sarin A, Mathur A. A mobile app based solution for implementing tobacco free educational institutions (ToFEI) guidelines. Tob Induc Dis. 2021 Sep 2;19(1).
82. Pulvers K, Rice M, Stewart SL, Tong E. Tobacco Tracker: A New Tool to Support College Smoke and Tobacco Free Policies. Nicotine Tob Res. 2022;24(2):241–9. Available from: https://academic.oup.com/ntr/article/24/2/241/6398215
83. New online tool helps keep college campuses smoke and tobacco free | TRDRP [Internet]. Available from: https://www.trdrp.org/news/tobacco-tracker-support-healthier-campus.html [Accessed 5 Mar 2022]
84. Chatterjee N, Patil D, Kadam R, Fernandes G. Tobacco-free School Policy in Maharashtra, India: A Qualitative Exploration of Implementation Facilitators and Barriers. Heal Behav Policy Rev. 2019;5(3):24–35.
85. Successful Collaboration Reduces Tobacco Use in Minnesota - News & Events | health.gov [Internet]. Available from: https://health.gov/news/201903/successful-collaboration-reduces-tobacco-use-minnesota[Accessed 7 Mar 2022]
86. Robertson LA, Marsh L. Smoke-free policies in New Zealand public tertiary education institutions. Health Educ Res. 2015;30(2):347–58. Available from: https://academic.oup.com/her/article/30/2/347/701723

For peer review only

For peer review only - http://bmjopen.bmj.com/site/about/guidelines.xhtml

Supplementary Table: An overview of qualitative data collection method, participants and themes				
**Themes**	**Stakeholders at National level**	**Stakeholders at state level**	**Stakeholders at district level**	**Stakeholders at village level**
Existing adolescent specific tobacco control programmes to prevent tobacco uptake among adolescents	✓	✓	✓	
Existing adolescent specific tobacco control policies to prevent tobacco uptake among adolescents	✓	✓	✓	
Perception on extent of program and policy implementation	✓	✓	✓	✓
Perception on designing and implementing adolescent specific tobacco control policies	✓	✓	✓	✓
Barriers/gaps for successful implementation of adolescent specific tobacco control policies and programmes	✓	✓	✓	✓
Facilitators for successful implementation of adolescent specific tobacco control policies and programmes	✓	✓	✓	✓
Recommended strategies to prevent and reduce the use of tobacco among adolescents	✓	✓	✓	✓
Need for programme evaluation and research priorities	✓	✓	✓	

For peer review only - http://bmjopen.bmj.com/site/about/guidelines.xhtml

BMJ Publishing Group Limited (BMJ) disclaims all liability and responsibility arising from any reliance placed on this supplemental material which has been supplied by the author(s)

SRQR Reporting checklist for qualitative study.

Instructions to authors

Complete this checklist by entering the page numbers from your manuscript where readers will find each of the items listed below. SRQR reporting guidelines: O'Brien BC, Harris IB, Beckman TJ, Reed DA, Cook DA. Standards for reporting qualitative research: a synthesis of recommendations. Acad Med. 2014;89(9):1245-1251.

		Reporting Item	Page Number
**Title**			
	#1	Concise description of the nature and topic of the study identifying the study as qualitative or indicating the approach (e.g. ethnography, grounded theory) or data collection methods (e.g. interview, focus group) is recommended	Page 1
**Abstract**			Abstract
	#2	Summary of the key elements of the study using the abstract format of the intended publication; typically includes background, purpose, methods, results and conclusions	Page 3
**Introduction**			
Problem formulation	#3	Description and signifcance of the problem / phenomenon studied: review of relevant theory and empirical work; problem statement	Introduction Page 5-6
Purpose or research question	#4	Purpose of the study and specific objectives or questions	Introduction Page 6
**Methods**			
Qualitative approach and research paradigm	#5	Qualitative approach (e.g. ethnography, grounded theory, case study, phenomenolgy, narrative research) and guiding theory if appropriate; identifying the research paradigm (e.g. postpositivist, constructivist / interpretivist) is also recommended; rationale. The rationale should briefly discuss the justification for choosing that theory, approach, method or technique rather than other options available; the assumptions and limitations implicit in those choices and how those choices influence study conclusions and transferability. As appropriate the rationale for several items might be discussed together.	Recruitment and Sampling: Page 7<br>Data analysis: Page 8
Researcher characteristics and reflexivity	#6	Researchers' characteristics that may influence the research, including personal attributes, qualifications / experience, relationship with participants, assumptions and / or presuppositions; potential or actual interaction between researchers' characteristics and the research questions, approach, methods, results and / or transferability	Data collection and procedure: Page 7-8
Context	#7	Setting / site and salient contextual factors; rationale	Recruitment and Sampling: Page 7
Sampling strategy	#8	How and why research participants, documents, or events were selected; criteria for deciding when no further sampling was necessary (e.g. sampling saturation); rationale	Recruitment and sampling Page 7
Ethical issues pertaining to human subjects	#9	Documentation of approval by an appropriate ethics review board and participant consent, or explanation for lack thereof; other confidentiality and data security issues	Back Material; Page 21-22
Data collection methods	#10	Types of data collected; details of data collection procedures including (as appropriate) start and stop dates of data collection and analysis, iterative process, triangulation of sources / methods, and modification of procedures in response to evolving study findings; rationale	Data Collection and procedure Page 7
Data collection	#11	Description of instruments (e.g. interview guides,	Data collection and procedure: Page 7

For peer review only - http://bmjopen.bmj.com/site/about/guidelines.xhtml

instruments and technologies		questionnaires) and devices (e.g. audio recorders) used for data collection; if / how the instruments(s) changed over the course of the study	
Units of study	#12	Number and relevant characteristics of participants, documents, or events included in the study; level of participation (could be reported in results)	Results: Page 8
Data processing	#13	Methods for processing data prior to and during analysis, including transcription, data entry, data management and security, verification of data integrity, data coding, and anonymisation / deidentification of excerpts	Data analysis: Page 8
Data analysis	#14	Process by which inferences, themes, etc. were identified and developed, including the researchers involved in data analysis; usually references a specific paradigm or approach; rationale	Data analysis: Page 8
Techniques to enhance trustworthiness	#15	Techniques to enhance trustworthiness and credibility of data analysis (e.g. member checking, audit trail, triangulation); rationale	Data analysis: Page 8
**Results/findings**			
Syntheses and interpretation	#16	Main findings (e.g. interpretations, inferences, and themes); might include development of a theory or model, or integration with prior research or theory	Results: Page 9-16
Links to empirical data	#17	Evidence (e.g. quotes, field notes, text excerpts, photographs) to substantiate analytic findings	Results: Page 9-16
**Discussion**			
Intergration with prior work, implications, transferability and contribution(s) to the field	#18	Short summary of main findings; explanation of how findings and conclusions connect to, support, elaborate on, or challenge conclusions of earlier scholarship; discussion of scope of application / generalizability; identification of unique contributions(s) to scholarship in a discipline or field	Discussion: Page 16
Limitations	#19	Trustworthiness and limitations of findings	Discussion: Page 20
**Other**			
Conflicts of interest	#20	Potential sources of influence of perceived influence on study conduct and conclusions; how these were managed	Back materials Page 21
Funding	#21	Sources of funding and other support; role of funders in data collection, interpretation and reporting	Back materials Page 21

None The SRQR checklist is distributed with permission of Wolters Kluwer © 2014 by the Association of American Medical Colleges. This checklist can be completed online using https://www.goodreports.org/, a tool made by the EQUATOR Network in collaboration with Penelope.ai

For peer review only

Hennegan J, et al. BMJ Global Health 2020; 5:e003433. doi: 10.1136/bmjgh-2020-003433

BMJ Open

Programme and Policy Perspectives Towards a Tobacco-Free Generation in India: Findings from a Qualitative Study

Journal:	*BMJ Open*
Manuscript ID	bmjopen-2022-067779.R2
Article Type:	Original research
Date Submitted by the Author:	24-Mar-2023
Complete List of Authors:	Bassi, Shalini; Public Health Foundation of India, Health Promotion Division; HRIDAY,<br>Arora, Monika; HRIDAY; Public Health Foundation of India, Health Promotion Division<br>Thapliyal, Nishibha; Public Health Foundation of India, Health Promotion Division<br>Kulkarni, Muralidhar; Kasturba Medical College Manipal, Community Medicine<br>Bhagawath, Rohith; Kasturba Medical College Manipal, Community Medicine<br>Bogdanovica, Ilze; University of Nottingham, School of Medicine<br>Kamath, Veena; Kasturba Medical College Manipal, Community Medicine<br>Britton, John; University of Nottingham, Epidemiology and Public Health<br>Bains, Manpreet; University of Nottingham, School of Medicine
**Primary Subject Heading**:	Public health
Secondary Subject Heading:	Health policy, Smoking and tobacco
Keywords:	QUALITATIVE RESEARCH, PUBLIC HEALTH, Health policy < HEALTH SERVICES ADMINISTRATION & MANAGEMENT

SCHOLARONE™
Manuscripts

I, the Submitting Author has the right to grant and does grant on behalf of all authors of the Work (as defined in the below author licence), an exclusive licence and/or a non-exclusive licence for contributions from authors who are: i) UK Crown employees; ii) where BMJ has agreed a CC-BY licence shall apply, and/or iii) in accordance with the terms applicable for US Federal Government officers or employees acting as part of their official duties; on a worldwide, perpetual, irrevocable, royalty-free basis to BMJ Publishing Group Ltd ("BMJ") its licensees and where the relevant Journal is co-owned by BMJ to the co-owners of the Journal, to publish the Work in this journal and any other BMJ products and to exploit all rights, as set out in our licence.

The Submitting Author accepts and understands that any supply made under these terms is made by BMJ to the Submitting Author unless you are acting as an employee on behalf of your employer or a postgraduate student of an affiliated institution which is paying any applicable article publishing charge ("APC") for Open Access articles. Where the Submitting Author wishes to make the Work available on an Open Access basis (and intends to pay the relevant APC), the terms of reuse of such Open Access shall be governed by a Creative Commons licence – details of these licences and which Creative Commons licence will apply to this Work are set out in our licence referred to above.

Other than as permitted in any relevant BMJ Author's Self Archiving Policies, I confirm this Work has not been accepted for publication elsewhere, is not being considered for publication elsewhere and does not duplicate material already published. I confirm all authors consent to publication of this Work and authorise the granting of this licence.

For peer review only

Title: Programme and Policy Perspectives Towards a Tobacco-Free Generation in India: Findings from a Qualitative Study

Authors: Shalini Bassi[1,2]*, Monika Arora[1,2], Nishibha Thapliyal[2], Muralidhar M Kulkarni[3], Rohith Bhagawath[3], Ilze Bogdanovica[4], Veena G Kamath[3], John Britton[4], Manpreet Bains[4]

[1] Health Related Information Dissemination Amongst Youth (HRIDAY)

[2] Public Health Foundation of India (PHFI)

[3] Manipal Academy of Higher Education

[4] University of Nottingham

Corresponding Author*

Ms. Shalini Bassi,

Senior Research Scientist, Health Promotion Division, Public Health Foundation of India (PHFI), Gurgaon, Haryana, 122002, India. shalini.bassi@phfi.org

&

Consultant-HRIDAY

N-25, Second Floor, Green Park Extension, New Delhi – 110016, India. shalini@hriday-shan.org

Author Information

Ms. Shalini Bassi, Health Promotion Division, Public Health Foundation of India (PHFI), Gurugram, India. & HRIDAY, New Delhi, India. shalini.bassi@phfi.org

Dr. Monika Arora, HRIDAY, New Delhi, India & Health Promotion Division, Public Health Foundation of India (PHFI), Gurugram, Haryana, India. monika@hriday-shan.org

For peer review only

Dr. Nishibha Thapliyal, Health Promotion Division, Public Health Foundation of India (PHFI), Gurugram, India, nishibhathapliyal@gmail.com

Dr. Muralidhar M Kulkarni, Community Medicine, Kasturba Medical College Manipal, Udupi, Karnataka, India. murali.kulkarni@manipal.edu

Dr. Veena Ganesh Kamath, Community Medicine, Kasturba Medical College Manipal, Udupi, Karnataka, India. veenak@manipal.edu

Dr. Rohith Bhagawath, Community Medicine, Kasturba Medical College Manipal, Manipal Academy of Higher Education, Udupi, Karnataka, India. rohit.bhagawath@manipal.edu

Dr. Ilze Bogdanovica, School of Medicine, University of Nottingham, Nottingham, United Kingdom. Ilze.Bogdanovica@nottingham.ac.uk

Prof. John Britton, Emeritus Professor; Director, UK Centre for Tobacco & Alcohol Studies, University of Nottingham, Division of Epidemiology and Public Health, Nottingham, United Kingdom. John.Britton1@nottingham.ac.uk; j.britton@outlook.com

Dr. Manpreet Bains, School of Medicine, University of Nottingham, Nottingham, United Kingdom. manpreet.bains@nottingham.ac.uk

Word Count – Abstract- 231 words; Main document- 4945

For peer review only - http://bmjopen.bmj.com/site/about/guidelines.xhtml

ABSTRACT

Objective: This study explored multi-stakeholder perspectives on existing adolescent-specific tobacco control policies and programmes, to advance India's transition towards a tobacco-free generation.

Design: Qualitative semi-structured interviews

Setting: Interviews were conducted with officials involved in tobacco control at the national (India), state (Karnataka), district (Udupi), and village level. Interviews were audio-recorded, transcribed verbatim, and analysed thematically.

Participants: Thirty-eight individuals representing national (n=9), state (n=9), district (n=14), and village (n=6) levels, participated.

Results: Themes highlighted the need to strengthen and amend the existing Tobacco Control Law (2003) provisions, particularly in the vicinity of schools (Sections 6a & 6b). Increasing the minimum legal age to buy tobacco from 18 to 21 years and developing an 'application' for 'compliance and monitoring indicators' in ToFEI guidelines were proposed. Policies to address smokeless tobacco use, and for stricter enforcement including regular monitoring of existing programmes, and robust evaluation of policies was underscored. Engaging adolescents to co-create interventions was advocated, along with integrating national tobacco control programmes into existing school and adolescent health programmes, utilizing both an inter-sectoral and whole-societal approach to prevent tobacco use, were recommended. Finally, stakeholders, mentioned that when drafting and implementing a comprehensive national tobacco control policy, there is a need to adopt a vision striving toward a tobacco-free generation.

Conclusion: Strengthening and developing tobacco control programmes and policies are warranted which are monitored and evaluated rigorously, and where adolescents should be involved, accordingly.

For peer review only

Strengths and limitations of this study

- This study captures perspectives on existing tobacco control policies in India, from a diverse range of stakeholders representing different levels of governance (national/state/district/village).
- Perspectives were captured on existing tobacco control policies and programmes, barriers, and facilitators to their successful implementation and suggested recommendations for strengthening existing and developing new policies to achieve the ultimate goal of a tobacco-free generation for India as well as replicate such models globally.
- Despite the fact that the interviewees for the in-depth interviews were not randomly selected, our study findings are still significant and transferable.

BACKGROUND

Tobacco use is a leading risk factor associated with several diseases, premature death, and disability. It is a major threat to developing and low/middle-income countries such as India, where tobacco use results in 1.35 million deaths, annually.[1,2] Furthermore, of all deaths related to non-communicable diseases in India, in 2019, approximately 1.08 million deaths (17.7%) were due to tobacco use.[3] The prevalence of tobacco use among adults in India has declined from 34% to 28% between 2010 (GATS-1)[4] and 2017 (GATS-2)[5] and among school-going students aged 13-15 years from 16.9% (GYTS-1, 2003)[6] to 8.5% (GYTS-4, 2019).[7] The majority of adult tobacco users initiate use during adolescence,[8] often leading to lifelong use, with low quit rates.[9] Therefore, prevention of tobacco uptake among children and adolescents is crucial to reducing the harms caused by tobacco use. There are many tobacco control programmes and policies, but the need for age-specific policies has gathered prominence in recent decades. Adolescent-specific policies are warranted,

For peer review only

For peer review only - http://bmjopen.bmj.com/site/about/guidelines.xhtml

especially, preventing uptake in the first place.[10]

The Government of India (GoI) has implemented various policies and programmes to prevent tobacco uptake among children and adolescents. Section 6a of the Cigarettes and Other Tobacco Products (Prohibition of Advertisement and Regulation of Trade and Commerce, Production, Supply, and Distribution) Act (COTPA) 2003[11], stipulates the prohibition of the sale of tobacco products to and by minors; and Section 6b, prohibits the sale of tobacco products within the surrounding 100 yards of educational institutions.[11] As part of the 'National Tobacco Control Programme'(NTCP), India also launched the School Programme across public and private schools, to equip young people with the knowledge and appropriate skills to make informed decisions related to tobacco consumption and build understanding on the consequences of tobacco use.[12] To reinforce such efforts, the Ministry of Health and Family Welfare (MoHFW)-Government of India also introduced 'Tobacco-Free Educational Institution' (ToFEI) guidelines[13], for all education establishments, across India, to generate awareness about the harmful and long-term effects of tobacco use and to create healthy tobacco-free educational institutes.

Despite these efforts, the implementation of tobacco control policies continues to be a challenge.[14] The evidence is mixed on whether investing in youth and adolescent tobacco control and prevention efforts is effective.[15] A growing focus is on 'anti-tobacco' interest groups and their influence on policy implementation and success.[16] Multiple partnerships have been found to be beneficial in tobacco control policy implementation at various levels.[17,18] Thus, considering the perspectives of various stakeholders for formulating tobacco control policies might be the way ahead. Perspectives from multiple stakeholders increases the range and quantity of information for researchers and thus increases the likelihood of actions being developed and implemented. [19]

This study explored the views of a diverse range of stakeholders (including policy-makers, policy-implementers, representatives from civil society, implementation agencies, and other stakeholders) on India's existing tobacco control policies and programmes focusing on adolescents, barriers to and facilitators for their successful implementation, and suggested recommendations for the future.

METHODS

Design

Qualitative, one-to-one, semi-structured interviews were conducted with key officials at the national (India), state (Karnataka), district (Udupi), and village level.

Patient and Public Involvement

Patients or the public were not involved in the design, conduct, reporting, or dissemination plans of our research.

Recruitment and sampling

A purposive sampling strategy was developed[19,20] where a list of 51 eligible individuals was drafted by members of the research team (MA, SB, MK), based on professional networks and level of engagement with tobacco control programmes, at different levels of governance. The interviewees varied in terms of their roles, responsibilities, and seniority, to ensure that a wide range of perspectives were captured. Potential interviewees were approached by SB and RB via e-mail and telephone. A study information sheet and consent form were sent prior to completing interviews, and an interview was arranged at a time convenient for each individual.

For peer review only

For peer review only - http://bmjopen.bmj.com/site/about/guidelines.xhtml

Data collection and procedure

A semi-structured interview guide was developed to explore interviewee's views on existing adolescent-specific tobacco control policies and programmes in India, barriers and faciliators to successful implementation, as well as recommendations for their successful implementation and strengthening, to advance India's transition towards a tobacco-free generation. The interview guide was developed by (SB, MB, with inputs from MA, MK, VK) based on the School Programme component delivered as part of NTCP[12], ToFEI guidelines by MoHFW[13], provisions of the Indian Tobacco Control Act (2003)[11], that specifically addressed the prevention of tobacco uptake and use among children and adolescents (Supplementary Table 1).

Interviews were conducted between July 2019 and September 2021, in English by trained qualitative researchers. National and state-level stakeholder interviews were moderated by SB, while district and village level by MK and RB. Due to the COVID-19 mitigation measures in India (e.g., travel restrictions), national and state-level interviews were conducted virtually using Zoom (during December 2020-September 2021), while district and village level were conducted in-person (from July 2019 -March 2020). The interviewees were asked to review and sign a consent form prior to interview (verbal consent was sought for virtual interviews). The consent process covered permission for data collection, audio-recording of interviews, data to be used in research publications, and dissemination whilst maintaining interviewee confidentiality and anonymity.

Data analysis

Interviews were transcribed verbatim by members of the research team. Identifiable data were anonymized and double-checked for accuracy by senior members of the research team. Transcripts were analysed by SB, NT and MB using thematic analysis[21] where both inductive and deductive

For peer review only - http://bmjopen.bmj.com/site/about/guidelines.xhtml

approaches[22] were adopted. To ensure the validity of interpretations[23], each individual familiarized themselves with the data separately which led to the identification of initial codes. Further readings resulted in the development of more substantive themes and sub-themes. Following this, a series of virtual calls were organized between SB, NT, and MB to discuss preliminary themes which led to a more definitive thematic framework, which was agreed upon and then applied to the remaining data (by NT). NVivo 12.0 was used to facilitate the analysis process and subsequent synthesis and reporting.

RESULTS

Thirty-eight interviews were conducted with: 9 national-level, 9 state-level (Bengaluru, Karnataka), 14 district (Udupi) level, and 6 village-level stakeholders. (Table 1) Interviewees included officials from the NTCP, members of the tobacco control committee (national, state, and district level), representatives from civil society organizations working in tobacco control, health, education, agriculture, food and civil supplies, police, parliamentary affairs, district administration, rural development and Panchayati raj (a system of local self-government of villages in rural India). Interviews averaged, 44 minutes (range 20 to 111 minutes).

Table 1: Demographic characteristics of the study interviewees (N=38)

	N (%)
**Sex**	
Male	26(68.4%)
Female	12 (31.6%)
**Geographical representation**	
National	9 (23.7%)
State	9 (23.7%)
District	14 (36.8%)
Village level	6 (15.8%)
**Years of working experience in tobacco control**	
0-5 years	9 (23.7%)
6-10 years	7 (18.4%)

For peer review only

>10 years	10 (26.3%)
Not available	12 (31.6%)
**Type of organization**	
Government	26 (68.4%)
Civil society representatives	11 (29%)
Development Agency	1 (2.6%)

Thematic analysis

Four themes were generated: 1. Views on existing tobacco control policies and programmes focusing on adolescents in India; 2. Barriers and facilitators to successful implementation; 3. Recommendations for successful implementation, and 4. Strengthening adolescent-specific tobacco control policies and programmes to advance India's transition towards a tobacco-free generation.

Views on existing tobacco control policy and programme focusing on adolescents in India

Interviewees reported many tobacco control programmes and policies to prevent tobacco uptake among adolescents. They believed school-based programmes or interventions to have an important role in preventing adolescents from using tobacco. Most of the interviewees spoke about the 'School Health Programme', which forms part of NTCP, and a few also mentioned smaller regional or local initiatives such as, the 'Yellow line campaign' (wherein, two yellow lines are painted 100 yards away from the boundary wall of an education institute demarking a 'No Tobacco Zone'); the 'Rose Campaign' (where a rose flower was given to petty shop owners selling tobacco and were made aware of its consequences and asked to display boards with tobacco warnings); the 'National Population Education Project' (creating awareness or helping adolescents understand the impact of using tobacco through several activities, such as poster competitions, memes, folk dance, skits, role play, mono acting, etc) and also other community-based activities with the involvement of local

For peer review only - http://bmjopen.bmj.com/site/about/guidelines.xhtml

leaders and the formation of a 'Taluka level coordination committee' (a group of several villages counted together for administrative purposes), for controlling tobacco in the community.

As part of the NTCP, the interviewees mentioned the ongoing sensitization activities (sessions/talks/audio-visual clips) school children were exposed to, as well as the formation of anti-tobacco school committees, making an anti-tobacco pledge in school assembly, the organization of anti-tobacco themed competitions like drawing, posters, painting, quiz competitions, and relay-races. Some spoke of annual competitions to formulate an anti-tobacco message and role-play activities, along with cultural-day programmes in schools, to generate awareness of tobacco, among both students and parents. A representative from the State Education department mentioned that many school teachers are also invited to be a part of Government-organised sensitization training, which aims to create awareness about the consequences of using tobacco for school children, where manuals were reportedly provided to school authorities, to aid the process.

> *"We have received a manual, wherein information about cigarette content, and activities to be taken by the students to not fall into the trap is provided. There are many pictures in that, wherein it is displayed about the effects of usage of tobacco, and many more things are there in the manual. There are several activities and content that create awareness among students and we have already passed this to school authorities"*

Many interviewees also discussed the ToFEI guidelines developed by the Ministry of Health and Family Welfare. A national-level NGO representative highlighted that the most important indicator in ToFEI, deals with the implementation of section 6b of COTPA around the prohibition of the sale of tobacco near educational institutes:

For peer review only - http://bmjopen.bmj.com/site/about/guidelines.xhtml

"One of the great outcomes that have come out of this ToFEI programme is section 6b of COTPA which prohibits any shop selling tobacco within 100 yards of any educational institute, right? And the industry makes sure that the shops are there. One of the components in these guidelines makes us report the number of tobacco shops which are around these schools."

Barriers and facilitators to successful implementation of tobacco control programmes and policies

Most interviewees reported weak monitoring and enforcement of 'NTCP' and 'ToFEI guidelines' as the primary barrier to their successful implementation. A national level civil society representative stated that, "*there is no mechanism to monitor how many schools are tobacco-free in the state"*. Another interviewee mentioned that the guidelines were limited in terms of their reach, "*The guidelines are not reaching upto where it has to, like if we talk about educational institutions. The Principals, the administrative authorities of these educational institutions seem to be unaware and they are unaware because nobody has reached out to them"*. Some also referred to a lack of dedicated staff to carry out programme activities and budget as barriers to programme implementation. A national-level representative said,*"There are 75 districts, there is staff only in 26 districts, 26 districts do proper reporting and monitoring but the remaining districts are not able to do because there are no staff specific to our department."*

Some even mentioned the lack of sub-national level representation during the policy development process as a barrier. Many stated a lack of awareness about the ongoing nature of these programmes, the tobacco control act, and its provisions among members of

For peer review only - http://bmjopen.bmj.com/site/about/guidelines.xhtml

enforcement agencies (organizations that are responsible for programme implementation- education, police department, etc.), were barriers to successful implementation of programmes. The COVID-19 pandemic was also mentioned by some as a barrier, because of the way it has overwhelmed many systems, especially within health and education, due to schools being closed for more than one academic year making tobacco control trivial.

Many interviewees proposed a bottom-up approach to facilitate proper implementation of existing programmes and policies. Some suggested prioritizing the NTCP programme at all levels, especially sub-nationally, along with amalgamating school-based programmes under one umbrella, to facilitate better implementation of the NTCP. Some even mentioned implementing programmes (like NTCP and ToFEI guidelines) in coordination with other ministries, especially the police and education departments, for better coverage and enforcement. A national representative proposed a partnership between the Ministries of health and education to facilitate implementation of the ToFEI guidelines. Some representatives also mentioned that tobacco control needs to be incorporated and discussed in state and district-level developmental meetings. A state-level government representative said, *"District level Coordination Committee" meeting would be a very good platform where such issues can be highlighted and brought to notice of the deputy commissioner himself and when he pitches in."*

Recommendations for successful implementation of tobacco control policies and programmes

Awareness generation and capacity building of enforcement agencies

While talking about the lessons and recommendations for strengthening adolescent-specific tobacco control efforts, most interviewees underlined the need to continuously educate adolescents about the consequences of using tobacco and how the tobacco industry uses deceptive tactics to entice

For peer review only - http://bmjopen.bmj.com/site/about/guidelines.xhtml

them to take up their products. Using explicit examples, such as the techniques used by the tobacco industry were highlighted. More use of IEC (Information, Education, Communication) material, videos, short films, and awareness campaigns, were suggested, along with the use of social media platforms (*Facebook, Twitter, WhatsApp),* due to their popularity among the younger generation. Many stressed the need for targeted sensitization programmes for enforcement agencies on the importance of enforcing tobacco control laws.

Interviewees believed school-based interventions to be an important way to prevent adolescents from using tobacco. A state representative said to "*Take up important aspects of tobacco education into the curriculum of students wherein they are taught actively that, these are habits that are detrimentally harmful, and the industry is going to try and fool them and to look for industry interference*". Some interviewees also suggested co-creating adolescent-driven projects or interventions[24] engaging them in creating awareness among their peers, where an example from a school-based intervention delivered in Delhi schools was referred to:

> "*The MYTRI trial, "if you see this element of peer activism in terms of interventions. So, if you do school-based interventions which are actively involving youth, as a change maker or as advocates, those interventions are more effective"*.

Strengthening the enforcement and monitoring of existing tobacco control policies and programmes

A state civil society representative suggested the education department needed to develop a monitoring tool to ensure compliance with ToFEI guidelines.

> "*The educational department has to come up with a monitoring tool to assess the level of compliance with* ToFEI. *Practically someone from the education department has to physically verify the compliance. The school may upload the*

photographs but the physical verification by the concerned officers of the educational department and sharing the monthly progress report with the key stakeholders is more helpful to understand the level of compliance."

A national-level interviewee pointed out that the ToFEI guidelines need to be implemented properly, especially in village schools, where teachers must first be oriented, and better collaboration between ministries needs to be addressed. In rural areas, integrating ToFEI guidelines in 'Aadarsh gaon Yojana'(Model village, Rural Development Model)[25] which focuses on village development and includes social, cultural development, and social mobilization of the village community; or Clean India Mission (Swaach Bharat Abhiyan)[26] a countrywide campaign initiated by the Government of India to eliminate open defecation and improve solid waste management, was suggested.

To reduce and prevent tobacco use among youth, most interviewees emphasized establishing a comprehensive enforcement mechanism for implementing the COTPA Act, in the vicinity of educational institutions. Many even stated that specific amendments to COTPA-2003, especially surrounding educational institutes, such as increasing penalties/fines, were needed. The need for strengthening implementation of 6a and 6b of COTPA-2003 was highlighted by a state level Civil Society representative, "*So we have COTPA section 6a, and 6b provision. It says tobacco cannot be sold within 100 yards of any educational institution, so if you implement this particular section 100% in any state, any geographical area, then no chance of accessibility or availability of tobacco products*". Including section 6b in the 'regular checklist' for the school's inspector, along with strict enforcement in and around schools, was also advocated.

For peer review only - http://bmjopen.bmj.com/site/about/guidelines.xhtml

The significance of robust regular monitoring at every level was underscored by interviewees. All tobacco-related programmes, particularly those in schools, must be monitored by the government. The school's involvement in monitoring, was considered critical. An official from the health department said "*we conduct a programme in one school and go back to that school after 4 years, no change would be seen. So there need for proper monitoring of every school. So, there are 3000-4000 schools in every district. Covering all schools at such large scales can only be done through district human resources"*. Similarly, many interviewees also suggested engaging civil societies, student self-help groups (like Bharat Scouts, National Cadet Corps), and police functionaries for monitoring and enforcement, to maximize effectiveness and increase programme accessibility.

Multi-sectoral and multi-stakeholder engagement

All interviewees commented on the successful implementation of any programme being dependent on having effective and efficient inter-sectoral collaborations, from national to grassroot levels (Panchayati Raj Institutions at the village level). The active involvement of all stakeholders, including health, non-health, and NGOs, to safeguard adolescents from tobacco was also highlighted, "*we need to wage a battle. Finance, human resources, NGOs, policy, intersectoral collaboration not just within the health sector but with other departments also***"**. Many interviewees suggested integrating the NTCP into existing health and adolescent health programmes like the Ayushman Bharat Scheme[27], National Adolescent Health Programme (Rashtriya Kishor Swasthya Karyakram)[28] and, National Programme for Prevention and Control of Cancer, Diabetes, Cardiovascular Diseases and Stroke (NPCDCS).[29]

"We have a national tobacco control programme, we have a district cancer control programme, we have oral health programme, multiple tobacco control programmes which are being implemented. We have district tobacco control programme. There are multiple overlapping issues with each of

For peer review only - http://bmjopen.bmj.com/site/about/guidelines.xhtml

these programmes, there has to be coordinated way and mechanism in which this could be implemented at the ground level and there has to be consistency in the way in which it has to be implemented".

Regular impact evaluation

Interviewees underscored the importance of routine programme and policy evaluation, particularly when considering the evolving nature of tobacco industry tactics to target adolescents. Research studies could help in identifying the loopholes or lacunae of the ongoing programmes by providing evidence and encouraging them to take corrective actions to improve them. It was stated that there is a need to identify opportunities and weaknesses in the context of regional, state, and district variations, and thus, a multi-pronged approach should be developed accordingly,*"We need to calibrate intervention, and policies as per the development of industry and science and we regularly need to undertake impact evaluation studies to understand what are the gaps what we have done"*

Strengthening adolescent-specific tobacco control policies and programmes to advance India's transition towards a tobacco-free generation

Development of new tobacco-free generation-centric policies

Many interviewees highlighted the need for new and achievable policies which focus on realising a tobacco-free generation. Considering the magnitude of smokeless tobacco use in India, many interviewees suggested, shift in focus of policy making, from only smoking to both smoking and smokeless tobacco. Many even suggested increasing the minimum legal age to buy tobacco from 18 to 21 years. Vendor licensing was also identified as a key strategy to reduce accessibility to tobacco. A stakeholder from the state said, *"What happens when a child goes to buy chocolate or cakes and sees lots of tobacco products also...so there is a chance he may buy tobacco products*

For peer review only

also. Because everything is sold in one shop so there is a risk of a small child getting access to a tobacco shop. So here, by vendor licensing, what we are doing is, we are preventing accessibility of a child getting tobacco."

Interviewees also spoke about developing policies or regulations to prohibit tobacco advertising, promotion, and sale on 'Over The Top' (OTT) platforms. These are needed to protect minors from all direct and indirect methods (surrogate promotion by big tobacco) of promotion and sales on OTT platforms. E-cigarette sales on online platforms was referred to as an example of how compliance with the Prohibition of Electronic Cigarettes (Production, Manufacture, Import, Export, Transport, Sale, Distribution, Storage and Advertisement) Act, 2019 in the country is weak.[30] The interviewees stressed the need for strict monitoring of regulations on OTT platforms.

DISCUSSION

The study emphasised the need for educating adolescents, communities, teachers and enforcement agencies on the consequences of tobacco use, tobacco control laws, and the tobacco industry's deceptive tactics to target young people. For preventing tobacco use among adolescents, our study demonstrates the need to formulate policy around realising a tobacco-free generation, engaging adolescents meaningfully, co-creation of adolescent-specific tobacco control interventions, strengthening and amending COTPA provisions surrounding educational institutions, improving multi-sectoral and multi-stakeholder engagement, adopting the whole-societal approach, policies prioritizing smokeless tobacco control, and future research for identifying the loopholes or lacunae of the ongoing programmes. The importance of having robust monitoring and evaluation mechanisms was underscored, to ensure the most effective interventions and policies are in place.

For peer review only - http://bmjopen.bmj.com/site/about/guidelines.xhtml

The introduction of COTPA[11] and ToFEI guidelines[13] were considered prime steps towards tobacco control among adolescents. But even after years since these have been implemented, awareness and compliance remain a major concern, especially in rural settings and among enforcement agencies.[31–35] The study highlights a need to increase awareness among adolescents, teachers, community members, and law enforcement agencies, which is supported by previous research.[36–38] Evidence from Maharashtra (India) showcased the positive effects of sensitization efforts with police on enforcement of COTPA with an increased collection of 'challans'(fines)[39] and action against shops violating section 6b of COTPA.[38]

The need to develop adolescent-driven programmes to meaningfully engage adolescents, wherein they serve as ambassadors and be involved in creating awareness among their peers, their families, and the community about tobacco use, was repeatedly emphasised in our findings. Many successful youth driven tobacco-based initiatives[40–42] have observed favourable results in terms of policy development[43], sensitization and reduced current tobacco use among youth.[44] A campaign engaging National Service Scheme (NSS) volunteers from 540 colleges in Mumbai resulted in improving awareness of tobacco control issues in 176,000 students[45], and the use of NSS volunteers[46] was also suggested in our study to support tobacco control initiatives.

This study underscored the importance of using social media to promote tobacco control messages and restricting tobacco industry marketing on social media, due to its widespread use among youth.[47] Existing evidence supports the use of social media for tobacco control.[48–52] For example, a recent social media campaign sought to educate high school students on e-cigarettes led to greater knowledge and beliefs about the harmful effects of e-cigarette use, suggesting social media as a promising tool for tobacco education, among young people.[53] Another study also demonstrated the use of digital media to empower adolescents in smoking prevention.[54] The need for extending the

For peer review only - http://bmjopen.bmj.com/site/about/guidelines.xhtml

TAPS ban to cover OTT platforms was underscored in our study. There is ample evidence worldwide showcasing the use (direct and indirect) of social networking sites for TAPS specifically targeting youth.[55–58] By using social media influencers, tobacco giants have found a way to circumvent policy guidelines.[59] Singapore passed laws on cyber surveillance and social media monitoring to counter the tobacco menace[60] and has also penalized and taken legal action on the TAPS violation.[61] A comprehensive ban on TAPS to ensure online streaming platforms are compliant with COTPA, is crucial for India to protect children from exposure to tobacco products displayed on the OTT platforms.Our study also recognizes the need to amend existing policies and development of new policies from tobacco control and prevention to 'Tobacco-Free future Generation' or 'Tobacco Endgame', to make the next generation 'tobacco-free'. Several countries like Scotland[62], Singapore[63] New Zealand[64], Finland[65] and others are striving towards a tobacco-free goal. A study weighing up the impact of New Zealand's Tobacco-Free Generation policy found that the implementation of TFG results in a substantial decrease in smoking prevalence.[66] India also needs to undertake strategic steps towards a tobacco-free generation by coordinating ongoing efforts of implementing ToFEI guidelines and global youth campaigns like NMT21C (No More Tobacco in the 21st Century).[67]

India should also consider making policy changes in the minimum legal age to buy tobacco, raising it from 18 to 21 years, as suggested by the stakeholders in our study. The evidence shows a high percentage of smokeless (75%) and smoking (77%) tobacco users in India initiate use before or up to the age of 21 years.[68] This is also supported by the scientific evidence that the brain continues to develop until about the age of 25 years. During this time period, brain growth is not complete and susceptibility to the damaging effects of tobacco smoke may be enhanced.[69,70] The probability that adolescent will remain tobacco-free for the rest of his/her life is higher. The effectiveness of this

For peer review only - http://bmjopen.bmj.com/site/about/guidelines.xhtml

approach can be seen in California where 'Tobacco 21' was passed in 2016 and this law has led to a decrease in the illegal sale of tobacco[71,72] and a decline in cigarette smoking in high school students.[73]

India also faces the dual challenge of dealing with tobacco smoking and smokeless tobacco, especially in rural areas where the prevalence of smokeless tobacco use is much higher.[74] Additionally most smokeless tobacco users are dual users[75] (who use both smokeless and smoking tobacco). Furthermore, current tobacco laws in the country are oriented towards smoking tobacco. Hence, the need for the development of new policies to cater for smokeless tobacco users was also emphasized in both our and previous studies.[76] Vendor licensing as recommended in our study can help in reducing the vendor density, especially near schools[77], and lower the odds of cigarette use initiation in adolescents.[78]

Findings from our study prioritises a need to strengthen the school health component which forms part of the ongoing NTCP, as school settings are deemed best for tobacco use prevention and addiction for children, school staff, and families. Families have an enormous influence on a child's tobacco use behaviour[79,80] and this has also been reported in our study. Thus, parents, particularly in villages need to be sensitized and should be involved in school-based tobacco-free efforts, to reduce tobacco exposure at home.

In terms of stricter implementation, our findings also highlight COTPA section 6b as the most important indicator for tobacco control among adolescents. In line with our findings, evidence largely supports the stricter implementation of section 6b of COTPA decreased tobacco use in adolescents[81] and vice versa (increased tobacco use by adolescents to proximity and density of retail outlets near schools).[82] The ToFEI guidelines emphasized the Self-Evaluation Scorecard for Tobacco-Free Educational Institutions and this is well aligned with our study findings on the regular

For peer review only - http://bmjopen.bmj.com/site/about/guidelines.xhtml

evaluation of these guidelines.[13] With the widespread digital infrastructure in the country, compliance, and monitoring of the indicators of ToFEI guidelines could benefit from the use of mobile app-based systems as suggested by stakeholders in our study. Similar app-based solutions has been implemented in several Indian states[83] and globally[84,85] and has been reported to improve awareness and surveillance in and around schools.

Our study interviewees emphasized that improved enforcement and implementation require collaborative multi-sectoral partnerships, which include schools, parents, officials at all levels (national, state and district), local representatives, civil society, and government health and non-health departments. The multi-stakeholder partnerships have been observed to be beneficial in reducing tobacco use in many parts of the world.[17,86,87] For instance, in New Zealand almost all educational institutes that have declared themselves 100% tobacco-free reported collaboration with external providers.[88]

Despite the novel findings presented in the paper, a few study limitations are noted. We have captured the perspectives of a diverse range of stakeholders representing different levels of governance (at national, state, district, and village level) other than adolescents, thus the beneficiary perspective (adolescents) on tobacco control policies is missing. Sampling was one of the limitations of our study, as the interviewees for the in-depth interviews were purposely selected. Nevertheless, we had a fairly balanced response in terms of stakeholders from different levels of governance and years of experience in tobacco control making our study findings transferable and significant. Additionally, the lack of comparison of study findings to a framework can be looked at as a limitation that can be explored in future research.

CONCLUSION

For peer review only - http://bmjopen.bmj.com/site/about/guidelines.xhtml

This study provides recommendations for strengthening the enforcement of existing, and developing new tobacco control programmes and policies focusing on adolescents to advance India towards the ultimate goal of a tobacco-free future generation and replicate such models globally.

ACKNOWLEDGEMENT

The authors would like to acknowledge the contributions of Dr. Nishigandha Joshi for transcribing the data from in-depth interviews. The authors thank all the interviewees who participated in this research from renowned tobacco control organizations in India. The paper benefited significantly from their insights and cooperation.

AUTHOR'S CONTRIBUTORS

MA, JB, SB, MB, MMK developed the research questions and study methodology. SB, MB developed an interview guide and MA, MMK, VGK provided inputs to the interview guide. The list of interviewees was developed by MA, SB, MMK. SB, MMK, RB coordinated and conducted the interviews. Data was coded and thematically analysed by SB, MB & NT. SB, MB & NT drafted the manuscript. MA, JB, MMK, VGK, RB, IB critically reviewed the manuscript. All authors are responsible for the overall content as guarantor.

FUNDING STATEMENT

This research work is funded by the Medical Research Council of the United Kingdom under the Global Alliance for Chronic Lung diseases programme (MR/P008933/1) and was carried out as part of a collaborative project of the University of Nottingham, Manipal Academy of Higher Education, HRIDAY and the University of Bath.

For peer review only

COMPETING INTREST: N.A

ETHICAL APPROVAL STATEMENT:

The study was approved by the Indian Ministry of Health and Family Welfare Screening Committee (HMSC, 2017-0460), the Institutional Ethics Committee at the Centre for Chronic Disease Control (CCDC_IEC_11_2018), Manipal Academy of Higher Education (MAHE EC/012/2017) and the University of Nottingham, School of Medicine Ethics Committee (OVS200317).

DATA SHARING STATEMENT

Data are available on reasonable request. As per our Institutional data sharing policy, prior approval is required. After approval of the request, de-identified data can only be shared upon request.

REFERENCES

1. WHO. India loses 1% of its GDP to diseases and early deaths from tobacco use, finds WHO study. WHO. Published February 9, 2021. Accessed February 18, 2022. https://www.who.int/india/news/detail/09-02-2021-india-loses-1-of-its-gdp-to-diseases-and-early-deaths-from-tobacco-use-finds-who-study
2. Tobacco in India. Accessed February 18, 2022. https://www.who.int/india/health-topics/tobacco
3. GBD Results Tool | GHDx. Accessed February 18, 2022. http://ghdx.healthdata.org/gbd-results-tool
4. International Institute for Population Sciences (IIPS) and Ministry of Health and Family Welfare. Global Adult Tobacco Survey - GATS India 2009-10. Accessed February 18, 2022. https://ntcp.nhp.gov.in/assets/document/surveys-reports-publications/Global-Adult-Tobacco-Survey-India-2009-2010-Report.pdf
5. Tata Institute of Social Sciences (TISS). Global Adult Tobacco Survey GATS 2 India 2016-17. TISS and Ministry of Health and Family Welfare, Government of India., New Delhi. Published 2017. Accessed February 18, 2022. https://ntcp.nhp.gov.in/assets/document/surveys-reports-publications/Global-Adult-Tobacco-Survey-Second-Round-India-2016-2017.pdf
6. Tobacco Use Among Students And Teachers Findings from the Global Youth Tobacco Survey (GYTS) and Global School Personnel Survey (GSPS). Published online 2003.
7. International Institute for Population Sciences (IIPS) and Ministry of Health and Family Welfare. Global Youth Tobacco Survey GYTS-4 Factsheet. Accessed February 18, 2022.

For peer review only - http://bmjopen.bmj.com/site/about/guidelines.xhtml

https://ntcp.nhp.gov.in/assets/document/National_Fact_Sheet_of_fourth_round_of_Global_Youth_Tobacco_Survey_GYTS-4.pdf
8. Chadda R, Sengupta S. Tobacco use by Indian adolescents. *Tob Induc Dis*. 2002;1(2):111. doi:10.1186/1617-9625-1-2-111
9. Thakur D, Gupta A, Thakur A, Mazta SR, Sharma D. Prevalence of cigarette smoking and its predictors among school going adolescents of North India. *South Asian J Cancer*. 2014;3(4):193. doi:10.4103/2278-330X.142946
10. Ranabhat CL, Kim CB, Park MB, Jakovljevic M. Situation, impacts, and future challenges of tobacco control policies for youth: An explorative systematic policy review. *Front Pharmacol*. 2019;10:981. doi:10.3389/FPHAR.2019.00981/BIBTEX
11. Government of India. *COTPA 2003*.; 2003. Accessed April 8, 2022. https://legislative.gov.in/sites/default/files/A2003-34.pdf
12. National Tobacco Control Cell Ministry of Health and Family Welfare. Operational .pdf Operational Guidelines National Tobacco Control Programme National Tobacco Control - [PDF Document]. 2015. Accessed February 18, 2022. https://cupdf.com/document/operational-pdf-operational-guidelines-national-tobacco-control-programme-national.html
13. MoHFW. Guidelines for Tobacco Free Educational Institution (Revised). Accessed February 18, 2022. https://ntcp.nhp.gov.in/assets/document/TEFI-Guidelines.pdf
14. Kaur J, Jain DC. Tobacco Control Policies in India: Implementation and Challenges. *Indian J Public Health*. 2011;55(3):220. doi:10.4103/0019-557X.89941
15. Lantz PM, Jacobson PD, Warner KE, et al. Investing in youth tobacco control: a review of smoking prevention and control strategies. *Tob Control*. 2000;9(1):47-63. doi:10.1136/TC.9.1.47
16. Kuijpers TG, Kunst AE, Willemsen MC. Who calls the shots in tobacco control policy? Policy monopolies of pro and anti-tobacco interest groups across six European countries. *BMC Public Health*. 2019;19(1):1-13. doi:10.1186/S12889-019-7158-6/TABLES/3
17. Ravindra K, Tripathy JP, Tripathy NK. Dynamics of multi-stakeholder engagement and its role in achieving high compliance of a tobacco control programme. *World Dev Perspect*. 2016;3:7-11. doi:10.1016/J.WDP.2016.10.002
18. MacGregor S. Editorial: Ireland's workplace smoking ban. *http://dx.doi.org/101080/09687630412331330074*. 2009;12(SUPPL. 1):1-136. doi:10.1080/09687630412331330074
19. Patton MQ. *Qualitative Evaluation and Research Methods, 2nd Ed.* Sage Publications, Inc; 1990.
20. Emmel N. Sampling and Choosing Cases in Qualitative Research: A Realist Approach. Published online 2013. doi:10.4135/9781473913882
21. Braun V, Clarke V. Using thematic analysis in psychology. *Qual Res Psychol*. 2006;3(2):77-101. doi:10.1191/1478088706QP063OA
22. DeCarlo M. 6.3 Inductive and deductive reasoning. Published online August 7, 2018.
23. Patton MQ. Enhancing the quality and credibility of qualitative analysis. *Health Serv Res*. 1999;34(5 Pt 2):1189-1208.
24. Perry CL, Stigler MH, Arora M, Reddy KS. Preventing Tobacco Use Among Young People in India: Project MYTRI. *Am J Public Health*. 2009;99(5):899. doi:10.2105/AJPH.2008.145433
25. Government of India. Pradhan Mantri Adarsh Gram Yojana (PMAGY). 2018. Accessed February 18, 2022. https://pmagy.gov.in/Docs/PMAGY-Guidelines-07022019-H.pdf

For peer review only

26. Government of India. SwachhBharat- Swachh Bharat Abhiyan a nation-wide campaign in India. Accessed February 18, 2022. https://swachhbharat.mygov.in/
27. Ministry of Health and Family Welfare G of I. Ministry of Health and Family Welfare Government of India Operational Guidelines on School Health Programme under Ayushman Bharat Health and Wellness Ambassadors partnering to build a stronger future A Joint initiative of Ministry of Health & Family Welfa. Published online 2018.
28. Rashtriya Kishor Swasthya Karyakram (RKSK) | National Health Portal Of India. Accessed August 12, 2022. https://www.nhp.gov.in/rashtriya-kishor-swasthya-karyakram-rksk_pg
29. National Programme for prevention & Control of Cancer, Diabetes, Cardiovascular Diseases & stroke (NPCDCS) :: National Health Mission. Accessed April 8, 2022. https://www.nhm.gov.in/index1.php?lang=1&level=2&sublinkid=1048&lid=604
30. MINISTRY OF LAW AND JUSTICE. *THE PROHIBITION OF ELECTRONIC CIGARETTES (PRODUCTION, MANUFACTURE, IMPORT, EXPORT, TRANSPORT, SALE, DISTRIBUTION, STORAGE AND ADVERTISEMENT) ACT, 2019*.; 2019. Accessed February 20, 2022. https://ntcp.nhp.gov.in/assets/document/The-Prohibition-of-Electronic-Cigarettes-Production-Manufacture-Import-Export-Transport-Sale-Distribution-Storage-and-Advertisement)-Act-2019.pdf
31. Chaudhary A, Thakur A, Chauhan T, et al. Creation of a Smoke-free Environment for Children: An Assessment of Compliance to COTPA 2003 Legislation in an Urban Area. *Indian Pediatr 2019 5610*. 2019;56(10):837-840. doi:10.1007/S13312-019-1609-0
32. Pradhan A, Oswal K, Padhan A, et al. Cigarettes and Other Tobacco Products Act (COTPA) implementation in education institutions in India: A crosssectional study. *Tob Prev Cessat*. 2020;6(September):1-7. doi:10.18332/TPC/125722
33. Rath R, Krishnan A, Nongkynrih B, Misra P. Assessment of implementation status of Cigarettes and Other Tobacco Products Act (COTPA) and its awareness among residents in a block of Haryana. *Indian J Public Health*. 2018;62(2):100. doi:10.4103/IJPH.IJPH_126_17
34. Goel S, Kumar R, Lal P, Singh RJ. How effective is tobacco control enforcement to protect minors: Results from subnational surveys across four districts in India. *Int J Noncommunicable Dis*. 2016;1(3):116. doi:10.4103/2468-8827.198583
35. Ahuja N, Kathiresan J, Anand T, Isaakidis P, Bajaj D. I have heard about it for the first time from you! Implementation of tobacco control law by police personnel in India. *Public Heal Action*. 2019;8(4):194-201. doi:10.5588/PHA.18.0064
36. Rawat R, Gouda J, Shekhar C. Smokeless tobacco use among adult males in India and selected states: Assessment of education and occupation linkages. *http://dx.doi.org/101080/1091135920151083507*. 2015;26(2):236-246. doi:10.1080/10911359.2015.1083507
37. Doma Bhutia T, Lad N. Empowering teachers to implement tobacco free school campaigns in schools. *Tob Induc Dis*. 2019;17(1). doi:10.18332/TID/111535
38. Lad N, Patil D, Pilankar A, Kadam R. Advocacy with police department for effective implementation of tobacco control polices. *Tob Induc Dis*. 2018;16(1). doi:10.18332/TID/84163
39. Shinde DB. Advocacies creates champions for tobacco control through COTPA implementation & achieving sustainable development goal. *Tob Induc Dis*. 2021;19(1). doi:10.18332/TID/140942
40. SWATFlorida - Students Working Against Tobacco. Accessed March 7, 2022. https://www.swatflorida.com/

For peer review only - http://bmjopen.bmj.com/site/about/guidelines.xhtml

41. Youth Initiatives - Campaign for Tobacco-Free Kids. Accessed February 20, 2022. https://www.tobaccofreekids.org/what-we-do/youth-programs
42. Youth smoking and vaping prevention. Accessed March 7, 2022. https://truthinitiative.org/what-we-do/youth-smoking-prevention-education
43. Arora M, Stigler MH, Reddy KS. Effectiveness of health promotion in preventing tobacco use among adolescents in India research evidence informs the national tobacco control programme in India. *Glob Health Promot*. 2011;18(1):09-12. doi:10.1177/1757975910393163
44. Lisboa OC, Bernardes-Souza B, De Freitas Xavier LE, Almeida MR, Corrêa PCRP, Brinker TJ. A Smoking Prevention Program Delivered by Medical Students to Secondary Schools in Brazil Called "Education Against Tobacco": Randomized Controlled Trial. *J Med Internet Res 2019;21(2)e12854 https//www.jmir.org/2019/2/e12854*. 2019;21(2):e12854. doi:10.2196/12854
45. Mathur A, Seth S, Sarin A. Mobilizing National Service Scheme (NSS) Volunteers for Tobacco Control in India. *Tob Induc Dis*. 2021;19(1). doi:10.18332/TID/141285
46. Kumar P, Rastogi S, Sarin A. Motivating youth to mobilize community to work in tobacco control in Karnataka, India. *Tob Induc Dis*. 2021;19(1). doi:10.18332/TID/141287
47. Patterns of Internet Usage Among Youths In India. Accessed February 20, 2022. https://www.socialmediamatters.in/patterns-of-internet-usage-among-youths-in-india
48. Jawad M, Abass J, Hariri A, Akl EA. Social media use for public health campaigning in a low resource setting: The case of waterpipe tobacco smoking. *Biomed Res Int*. 2015;2015. doi:10.1155/2015/562586
49. Baskerville NB, Azagba S, Norman C, McKeown K, Brown KS. Effect of a digital social media campaign on young adult smoking cessation. *Nicotine Tob Res*. 2016;18(3):351-360. doi:10.1093/NTR/NTV119
50. U.S. DEPARTMENTOF HEALTH AND HUMAN SERVICES. *The Role of the Media in Promoting and Reducing Tobacco Use* . Accessed April 8, 2022. https://cancercontrol.cancer.gov/sites/default/files/2020-08/m19_complete.pdf
51. Luo T, Li MS, Williams D, et al. Using social media for smoking cessation interventions: a systematic review. *Perspect Public Health*. 2021;141(1):50-63. doi:10.1177/1757913920906845
52. Naslund JA, Kim SJ, Aschbrenner KA, et al. Systematic review of social media interventions for smoking cessation. *Addict Behav*. 2017;73:81-93. doi:10.1016/J.ADDBEH.2017.05.002
53. Lazard AJ. Social Media Message Designs to Educate Adolescents About E-Cigarettes. *J Adolesc Heal*. 2021;68(1):130-137. doi:10.1016/J.JADOHEALTH.2020.05.030
54. Park E, Chang YP. Using Digital Media to Empower Adolescents in Smoking Prevention: Mixed Methods Study. *JMIR Pediatr Parent*. 2020;3(1). doi:10.2196/13031
55. Jackler RK, Li VY, Cardiff RAL, Ramamurthi D. Promotion of tobacco products on Facebook: policy versus practice. *Tob Control*. 2019;28(1):67-73. doi:10.1136/TOBACCOCONTROL-2017-054175
56. Freeman B, Watts C, Ayu P, Astuti S. Global tobacco advertising, promotion and sponsorship regulation: what's old, what's new and where to next? *Tob Control*. 2022;31(2):216-221. doi:10.1136/TOBACCOCONTROL-2021-056551
57. Clendennen SL, Vandewater EA, Loukas A, Perry CL, Wilkinson A V. College students' exposure and engagement with tobacco-related social media. *Tob Regul Sci*. 2020;6(1):38-53. doi:10.18001/TRS.6.1.5

For peer review only - http://bmjopen.bmj.com/site/about/guidelines.xhtml

58. FreemanBecky. Big tobacco uses social media to spread cancer | UICC. UICC global cancer control. Published March 2, 2021. Accessed February 20, 2022. https://www.uicc.org/blog/big-tobacco-uses-social-media-spread-cancer#
59. Mutmainnah N, Hendriyani H, Utaminingtyas I. Outsmarting regulation: how tobacco websites and social media targeting young people. *Int J Commun Soc*. 2020;2(1):12-19. doi:10.31763/IJCS.V2I1.108
60. Lee T. Advanced country practices in the implementation of WHO FCTC Article 13 and its guidelines Combatting cross-border advertising and depiction of tobacco in entertainment media. Published online 2018.
61. 13 fined for illegal online sale of e-vaporisers, including one jailed for selling unregistered medicines - CNA. Accessed February 21, 2022. https://www.channelnewsasia.com/singapore/vape-e-vaporisers-sale-cough-syrup-sleeping-pills-2076111
62. Scottish Government T. CREATING A TOBACCO-FREE GENERATION A Tobacco Control Strategy for Scotland CREATING A TOBACCO-FREE GENERATION A Tobacco Control Strategy for Scotland The Scottish Government, Edinburgh 2013 ii-CREATING A TOBACCO-FREE GENERATION: A TOBACCO CONTROl STRATEGy FOR SCOTlANd.
63. Khoo D, Chiam Y, Ng P, Berrick AJ, Koong HN. Phasing-out tobacco: proposal to deny access to tobacco for those born from 2000. doi:10.1136/tc.2009.031153
64. New Zealand aims for smoke-free generation with new tobacco law | The Straits Times. Accessed March 7, 2022. https://www.straitstimes.com/asia/australianz/new-zealand-aims-for-smoke-free-generation-with-new-tobacco-law
65. Tobacco-free Finland 2030. Accessed April 8, 2022. https://savutonsuomi.fi/en/
66. van der Deen FS, Wilson N, Cleghorn CL, et al. Impact of five tobacco endgame strategies on future smoking prevalence, population health and health system costs: two modelling studies to inform the tobacco endgame. *Tob Control*. 2018;27(3):278-286. doi:10.1136/TOBACCOCONTROL-2016-053585
67. International Conference on Public Health Priorities in the 21st Century: The Endgame for Tobacco – Voice of Tobacco Victims. Accessed March 2, 2022. https://vovindia.org/2013/05/23/international-conference-public-health-priorities-21st-century-endgame-tobacco/
68. Lal P, Kapoor S, Dhankhar A, Yadav A. Raising the legal age for purchase and use of tobacco to 21 years: A step towards tobacco endgame in India. *Indian J Tuberc*. 2021;68:S65-S70. doi:10.1016/J.IJTB.2021.07.019
69. Bonnie RJ, Stratton K, Kwan LY, Products C on the PHI of R the MA for PT, Practice B on PH and PH, Medicine I of. *Public Health Implications of Raising the Minimum Age of Legal Access to Tobacco Products*. National Academies Press (US); 2015. doi:10.17226/18997
70. Understanding the Teen Brain - Health Encyclopedia - University of Rochester Medical Center. Accessed December 30, 2022. https://www.urmc.rochester.edu/encyclopedia/content.aspx?ContentTypeID=1&ContentID=3051
71. Zhang X, Vuong TD, Andersen-Rodgers E, Roeseler A. Evaluation of California's 'Tobacco 21' law. *Tob Control*. 2018;27(6):656-662. doi:10.1136/TOBACCOCONTROL-2017-054088
72. Ahmad S. The Cost-Effectiveness of Raising the Legal Smoking Age in California. *Med*

For peer review only - http://bmjopen.bmj.com/site/about/guidelines.xhtml

Decis Mak. 2005;25(3):330-340. doi:10.1177/0272989x05276859
73. CTFK. *INCREASING THE MINIMUM LEGAL SALE AGE FOR TOBACCO PRODUCTS TO 21*. Accessed March 26, 2022. https://www.tobaccofreekids.org/assets/factsheets/0376.pdf
74. Singh PK, Yadav A, Lal P, et al. Dual Burden of Smoked and Smokeless Tobacco Use in India, 2009–2017: A Repeated Cross-Sectional Analysis Based on Global Adult Tobacco Survey. *Nicotine Tob Res*. 2020;22(12):2196-2202. doi:10.1093/NTR/NTAA033
75. Noh JW, Kim MH, Lee Y, et al. Association between smokeless tobacco use and cigarette smoking amount by age. *BMC Public Heal 2022 221*. 2022;22(1):1-8. doi:10.1186/S12889-022-12929-Z
76. Yadav A, Singh PK, Yadav N, et al. Smokeless tobacco control in India: policy review and lessons for high-burden countries. *BMJ Glob Heal*. 2020;5(7):e002367. doi:10.1136/BMJGH-2020-002367
77. Pouranik NS, Saraf S, Wright K, et al. Tobacco retailer density and tobacco retailers near schools in two cities of East India, Ranchi and Siliguri. *Indian J Tuberc*. 2021;68:S14-S22. doi:10.1016/J.IJTB.2021.07.003
78. Astor RL, Urman R, Barrington-Trimis JL, et al. Tobacco retail licensing and youth product use. *Pediatrics*. 2019;143(2). doi:10.1542/PEDS.2017-3536/37292
79. CTFK. *HOW SCHOOLS CAN HELP STUDENTS STAY TOBACCO-FREE*. Accessed April 8, 2022. https://www.tobaccofreekids.org/assets/factsheets/0153.pdf
80. Adams ML, Jason LA, Pokorny S, Hunt Y. The Relationship Between School Policies and Youth Tobacco Use. *J Sch Health*. 2009;79(1):17. doi:10.1111/J.1746-1561.2008.00369.X
81. Mistry R, Pednekar MS, McCarthy WJ, et al. Compliance with point-of-sale tobacco control policies and student tobacco use in Mumbai, India. *Tob Control*. 2019;28(2):220-226. doi:10.1136/TOBACCOCONTROL-2018-054290
82. Henriksen L, Feighery EC, Schleicher NC, Cowling DW, Kline RS, Fortmann SP. Is adolescent smoking related to the density and proximity of tobacco outlets and retail cigarette advertising near schools? *Prev Med (Baltim)*. 2008;47(2):210-214. doi:10.1016/J.YPMED.2008.04.008
83. Seth S, Sarin A, Mathur A. A mobile app based solution for implementing tobacco free educational institutions (ToFEI) guidelines. *Tob Induc Dis*. 2021;19(1). doi:10.18332/TID/141286
84. Pulvers K, Rice M, Stewart SL, Tong E. Tobacco Tracker: A New Tool to Support College Smoke and Tobacco Free Policies. *Nicotine Tob Res*. 2022;24(2):241-249. doi:10.1093/NTR/NTAB187
85. New online tool helps keep college campuses smoke and tobacco free | TRDRP. Accessed March 5, 2022. https://www.trdrp.org/news/tobacco-tracker-support-healthier-campus.html
86. Chatterjee N, Patil D, Kadam R, Fernandes G. Tobacco-free School Policy in Maharashtra, India: A Qualitative Exploration of Implementation Facilitators and Barriers. *Heal Behav Policy Rev*. 2019;5(3):24-35. doi:10.14485/HBPR.5.3.3
87. Successful Collaboration Reduces Tobacco Use in Minnesota - News & Events | health.gov. Accessed March 7, 2022. https://health.gov/news/201903/successful-collaboration-reduces-tobacco-use-minnesota
88. Robertson LA, Marsh L. Smoke-free policies in New Zealand public tertiary education institutions. *Health Educ Res*. 2015;30(2):347-358. doi:10.1093/HER/CYV004

For peer review only

For peer review only - http://bmjopen.bmj.com/site/about/guidelines.xhtml

Supplementary Table: An overview of qualitative data collection method, participants and themes				
**Themes**	**Stakeholders at National level**	**Stakeholders at state level**	**Stakeholders at district level**	**Stakeholders at village level**
Existing adolescent specific tobacco control programmes to prevent tobacco uptake among adolescents	✓	✓	✓	
Existing adolescent specific tobacco control policies to prevent tobacco uptake among adolescents	✓	✓	✓	
Perception on extent of program and policy implementation	✓	✓	✓	✓
Perception on designing and implementing adolescent specific tobacco control policies	✓	✓	✓	✓
Barriers/gaps for successful implementation of adolescent specific tobacco control policies and programmes	✓	✓	✓	✓
Facilitators for successful implementation of adolescent specific tobacco control policies and programmes	✓	✓	✓	✓
Recommended strategies to prevent and reduce the use of tobacco among adolescents	✓	✓	✓	✓
Need for programme evaluation and research priorities	✓	✓	✓	

For peer review only - http://bmjopen.bmj.com/site/about/guidelines.xhtml

BMJ Publishing Group Limited (BMJ) disclaims all liability and responsibility arising from any reliance placed on this supplemental material which has been supplied by the author(s)

SRQR Reporting checklist for qualitative study.

Instructions to authors

Complete this checklist by entering the page numbers from your manuscript where readers will find each of the items listed below. SRQR reporting guidelines: O'Brien BC, Harris IB, Beckman TJ, Reed DA, Cook DA. Standards for reporting qualitative research: a synthesis of recommendations. Acad Med. 2014;89(9):1245-1251.

		Reporting Item	Page Number
**Title**			
	#1	Concise description of the nature and topic of the study identifying the study as qualitative or indicating the approach (e.g. ethnography, grounded theory) or data collection methods (e.g. interview, focus group) is recommended	Page 1
**Abstract**			Abstract
	#2	Summary of the key elements of the study using the abstract format of the intended publication; typically includes background, purpose, methods, results and conclusions	Page 3
**Introduction**			
Problem formulation	#3	Description and signifcance of the problem / phenomenon studied: review of relevant theory and empirical work; problem statement	Introduction Page 5-6
Purpose or research question	#4	Purpose of the study and specific objectives or questions	Introduction Page 6
**Methods**			
Qualitative approach and research paradigm	#5	Qualitative approach (e.g. ethnography, grounded theory, case study, phenomenolgy, narrative research) and guiding theory if appropriate; identifying the research paradigm (e.g. postpositivist, constructivist / interpretivist) is also recommended; rationale. The rationale should briefly discuss the justification for choosing that theory, approach, method or technique rather than other options available; the assumptions and limitations implicit in those choices and how those choices influence study conclusions and transferability. As appropriate the rationale for several items might be discussed together.	Recruitment and Sampling: Page 7<br>Data analysis: Page 8
Researcher characteristics and reflexivity	#6	Researchers' characteristics that may influence the research, including personal attributes, qualifications / experience, relationship with participants, assumptions and / or presuppositions; potential or actual interaction between researchers' characteristics and the research questions, approach, methods, results and / or transferability	Data collection and procedure: Page 7-8
Context	#7	Setting / site and salient contextual factors; rationale	Recruitment and Sampling: Page 7
Sampling strategy	#8	How and why research participants, documents, or events were selected; criteria for deciding when no further sampling was necessary (e.g. sampling saturation); rationale	Recruitment and sampling Page 7
Ethical issues pertaining to human subjects	#9	Documentation of approval by an appropriate ethics review board and participant consent, or explanation for lack thereof; other confidentiality and data security issues	Back Material; Page 21-22
Data collection methods	#10	Types of data collected; details of data collection procedures including (as appropriate) start and stop dates of data collection and analysis, iterative process, triangulation of sources / methods, and modification of procedures in response to evolving study findings; rationale	Data Collection and procedure Page 7
Data collection	#11	Description of instruments (e.g. interview guides,	Data collection and procedure: Page 7

For peer review only - http://bmjopen.bmj.com/site/about/guidelines.xhtml

instruments and technologies		questionnaires) and devices (e.g. audio recorders) used for data collection; if / how the instruments(s) changed over the course of the study	
Units of study	#12	Number and relevant characteristics of participants, documents, or events included in the study; level of participation (could be reported in results)	Results: Page 8
Data processing	#13	Methods for processing data prior to and during analysis, including transcription, data entry, data management and security, verification of data integrity, data coding, and anonymisation / deidentification of excerpts	Data analysis: Page 8
Data analysis	#14	Process by which inferences, themes, etc. were identified and developed, including the researchers involved in data analysis; usually references a specific paradigm or approach; rationale	Data analysis: Page 8
Techniques to enhance trustworthiness	#15	Techniques to enhance trustworthiness and credibility of data analysis (e.g. member checking, audit trail, triangulation); rationale	Data analysis: Page 8
**Results/findings**			
Syntheses and interpretation	#16	Main findings (e.g. interpretations, inferences, and themes); might include development of a theory or model, or integration with prior research or theory	Results: Page 9-16
Links to empirical data	#17	Evidence (e.g. quotes, field notes, text excerpts, photographs) to substantiate analytic findings	Results: Page 9-16
**Discussion**			
Intergration with prior work, implications, transferability and contribution(s) to the field	#18	Short summary of main findings; explanation of how findings and conclusions connect to, support, elaborate on, or challenge conclusions of earlier scholarship; discussion of scope of application / generalizability; identification of unique contributions(s) to scholarship in a discipline or field	Discussion: Page 16
Limitations	#19	Trustworthiness and limitations of findings	Discussion: Page 20
**Other**			
Conflicts of interest	#20	Potential sources of influence of perceived influence on study conduct and conclusions; how these were managed	Back materials Page 21
Funding	#21	Sources of funding and other support; role of funders in data collection, interpretation and reporting	Back materials Page 21

None The SRQR checklist is distributed with permission of Wolters Kluwer © 2014 by the Association of American Medical Colleges. This checklist can be completed online using https://www.goodreports.org/, a tool made by the EQUATOR Network in collaboration with Penelope.ai

For peer review only

Hennegan J, *et al. BMJ Global Health* 2020; 5:e003433. doi: 10.1136/bmjgh-2020-003433
